# Supplementary material for: Joint time-to-event partial order continual reassessment method and Joint time-to-event Bayesian logistic regression model: Statistical designs for dual agent phase I/II dose finding studies with late-onset toxicity and activity outcomes
Source: Stat Methods Med Res. 2025 Dec 5;35(1):186–204. doi: 10.1177/09622802251403384 (PMC13044421; doi:10.1177/09622802251403384)
Supplement: sj-pdf-1-smm-10.1177_09622802251403384 - Supplemental material for Joint time-to-event partial order continual reassessment method and Joint time-to-event Bayesian logistic regression model: Statistical designs for dual agent phase I/II dose finding studies with late-onset toxicity and activity outc [file sj-pdf-1-smm-10.1177_09622802251403384.pdf]

Joint TITE-POCRM and Joint TITE-BLRM: Statistical Designs for Dual Agent Phase I/II Dose Finding Studies with Late-Onset Toxicity and Activity Outcomes: Online Supplementary Materials

1 TITE-Comb-BOIN12

Here we give further details of the TITE-comb-BOIN12 [3] design that is used as a model-assisted comparator. This is an extension of the TITE-BOIN12 design [8] for single agent trials, using the dual agent BOIN design [2]. As a model-assisted design, a model is used at each dose level to make escalation decisions, rather than modelling the dose-response relationship like the two model-based designs. Further details can be found in the original proposal by Lu et al. (2024) [3].

The utility function used is as follows, for dose d_{ij}:

$$u_{i,j} = \hat{\pi_E}(d_{ij}) - W_T \hat{\pi_T}(d_{ij})$$

An approximated likelihood is used at each dose level, using x, the number of so-called *quasi-events*. Further details of this can be found in the original proposal [3]. The posterior distribution of the utility (u^*) at each dose is:

$$u^*|D \sim Beta(1 + x, 1 + n - x)$$

Since it is assumed $x \sim Bin(n, u^*)$ with prior $u^* \sim Beta(1, 1)$.

The utility criterion measure used in the dosing decision is $P(u^* > u_b|D)$, with the objective to choose the dose out of the defined set that maximises the probability of being above some utility threshold, u_b.

The de-escalation set $\mathcal{A}_D$, stay set $\mathcal{A}_S$ and escalation set $\mathcal{A}_E$ for dose combination d_{ij} are defined as:

$$\mathcal{A}_D = \{d_{i-1,j}, d_{i,j-1}\}$$

$$\mathcal{A}_S = \{d_{i-1,j}, d_{i,j-1}, d_{ij}\}$$

$$\mathcal{A}_E = \{d_{i-1,j}, d_{i,j-1}, d_{i+1,j-1}, d_{i-1,j+1}, d_{ij}, d_{i+1,j}, d_{i,j+1}\}$$

The choice of the next dose combination is then made at each dosing decision point in the following way. Consider the current dose as d_{ij}.

- If $\hat{\pi_T}(d_{ij}) \geq \lambda_d$, then the decision is made to de-escalate to the dose combination in $\mathcal{A}_D$ that is admissible and that maximises $P(u^* > u_b|D)$.
- If $\lambda_e < \hat{\pi_T}(d_{ij}) < \lambda_d$, then choose the dose combination in the set $\mathcal{A}_S$ that is admissible and that maximises $P(u^* > u_b|D)$.
- Otherwise, choose the dose combination in the set $\mathcal{A}_E$ that is admissible and that maximises $P(u^* > u_b|D)$.

This approach is labelled as 'Alternative BOIN Utility', with results given in Section 6. Results given in the main manuscript use the utility introduced in the manuscript, but all other aspects of the approach remain the same as outlined here.

2 Prior Distributions

Since all methods are Bayesian, we must specify prior distributions for the parameters of the models.

2.0.1 Prior Calibration

For both the Joint TITE-POCRM and the Joint TITE-BLRM, some of the hyper-parameters that specify the prior distribution are calibrated (see for example [4]) to give overall good performance in terms of Percentage of Correct Selections (PCS). The process of the calibration is as follows:

1. Consider only the safety model, and calibrate the safety part of the prior via a grid search over a number of plausible safety scenarios, choosing the combination of values giving the highest geometric mean PCS.

2. Consider the full joint model of safety and activity, fixing the prior for safety as chosen previously and calibrate the activity part of the prior, considering a number of reasonable settings over a number of safety/activity scenarios, choosing the combination of values giving the highest geometric mean PCS.

3. Consider the full joint model of safety and activity, fixing the prior for activity as chosen previously and calibrate the safety part of the prior, considering a number of reasonable settings over a number of safety/activity scenarios, choosing the combination of values giving the highest geometric mean PCS.

4. Repeat steps 2 and 3 until the improvement in PCS is below a pre-specified bound.

2.0.2 Joint TITE-POCRM

The skeletons for activity and toxicity are as follows
$x^{(A)} = (0.300, 0.402, 0.501, 0.593, 0.673, 0.741, 0.797, 0.842, 0.878, 0.906)$ and
$x^{(T)} = (0.261, 0.300, 0.340, 0.381, 0.422, 0.462, 0.501, 0.538, 0.574, 0.609)$

The priors used in Part 1, for the independent one-parameter models are in-line with the original POCRM [6], $\alpha^{(A)} \sim N(0, 1.34^2)$ and $\alpha^{(T)} \sim N(0, 1.34^2)$ The following prior is used in Part 2, in-line with the Joint-TITE-CRM [1]:

$$\begin{pmatrix} \beta_0 \\ \log(\beta_1) \end{pmatrix} \sim N_2 \left(\begin{pmatrix} c_1 \\ c_2 \end{pmatrix}, \begin{pmatrix} v_1 & 0 \\ 0 & v_2 \end{pmatrix} \right). \tag{1}$$

for both toxicity and activity. Calibrated via the procedure outline in Section 2.0.1, the values of hyper-parameters used are given in Table 1. We used a vague prior for $\psi \sim N(0, 100)$.

			c_1	c_2	v_1	v_2
Joint TITE-POCRM		Toxicity	-4	2.5	1	0.25
		Activity	-4	2	1.5	0.5
Joint TITE-BLRM	Agent W_1	Toxicity	-6	0	1	0.5
		Activity	-3	-5	1	4
	Agent W_2	Toxicity	-5.5	1	1	0.5
		Activity	-4	-5	1	4

Table 1: Values of hyperparameter for the Joint TITE-POCRM and Joint TITE-BLRM.

As well as prior distributions, the Joint TITE-POCRM also requires the specification of partial orderings for both activity and toxicity. The set of considered partial orderings is the same for both activity and toxicity. Here, the specifications of partial orderings follow those of Wages et al. [7]. For the 2×5 grid:

Order 1 (Rows):

$$R(d_{11}) \leq R(d_{12}) \leq R(d_{13}) \leq R(d_{14}) \leq R(d_{15}) \leq R(d_{21}) \leq R(d_{22}) \leq R(d_{23}) \leq R(d_{24}) \leq R(d_{25})$$

Order 2 (Columns/Up Diagonal):

$$R(d_{11}) \leq R(d_{21}) \leq R(d_{12}) \leq R(d_{22}) \leq R(d_{13}) \leq R(d_{23}) \leq R(d_{14}) \leq R(d_{24}) \leq R(d_{15}) \leq R(d_{25})$$

Order 3 (Up-down Diagonal):

$$R(d_{11}) \leq R(d_{12}) \leq R(d_{21}) \leq R(d_{13}) \leq R(d_{22}) \leq R(d_{14}) \leq R(d_{23}) \leq R(d_{15}) \leq R(d_{24}) \leq R(d_{25})$$

Order 4 (Down-up Diagonal):

$$R(d_{11}) \leq R(d_{12}) \leq R(d_{21}) \leq R(d_{22}) \leq R(d_{13}) \leq R(d_{14}) \leq R(d_{23}) \leq R(d_{24}) \leq R(d_{15}) \leq R(d_{25})$$

Order 5 (Down Diagonal):

$$R(d_{11}) \leq R(d_{21}) \leq R(d_{12}) \leq R(d_{13}) \leq R(d_{22}) \leq R(d_{14}) \leq R(d_{23}) \leq R(d_{15}) \leq R(d_{24}) \leq R(d_{25})$$

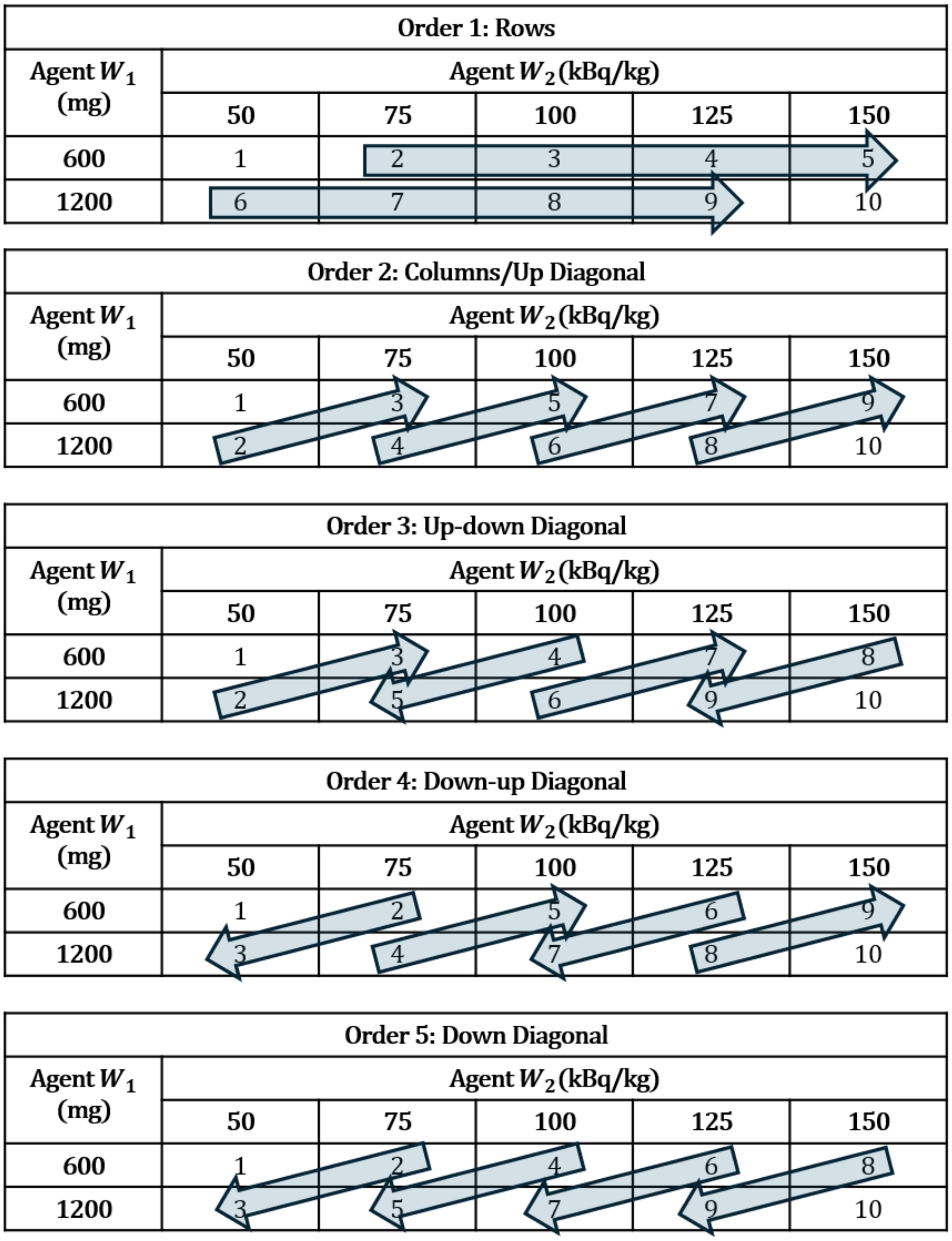

Figure 1: Visualisation of the five partial orderings considered in the 2×5 grid.

For the 3×3 grid:

Order 1 (Rows):

$$R(d_{11}) \leq R(d_{12}) \leq R(d_{13}) \leq R(d_{21}) \leq R(d_{22}) \leq R(d_{23}) \leq R(d_{31}) \leq R(d_{32}) \leq R(d_{33})$$

Order 2 (Columns):

$$R(d_{11}) \leq R(d_{21}) \leq R(d_{31}) \leq R(d_{12}) \leq R(d_{22}) \leq R(d_{32}) \leq R(d_{13}) \leq R(d_{23}) \leq R(d_{33})$$

Order 3 (Down-up Diagonal):

$$R(d_{11}) \leq R(d_{21}) \leq R(d_{12}) \leq R(d_{13}) \leq R(d_{22}) \leq R(d_{31}) \leq R(d_{32}) \leq R(d_{23}) \leq R(d_{33})$$

Order 4 (Up-down Diagonal):

$$R(d_{11}) \leq R(d_{12}) \leq R(d_{21}) \leq R(d_{31}) \leq R(d_{22}) \leq R(d_{13}) \leq R(d_{23}) \leq R(d_{32}) \leq R(d_{33})$$

Order 5 (Up Diagonal):

$$R(d_{11}) \leq R(d_{12}) \leq R(d_{21}) \leq R(d_{13}) \leq R(d_{22}) \leq R(d_{31}) \leq R(d_{23}) \leq R(d_{32}) \leq R(d_{33})$$

Order 6 (Down Diagonal):

$$R(d_{11}) \leq R(d_{21}) \leq R(d_{12}) \leq R(d_{31}) \leq R(d_{22}) \leq R(d_{13}) \leq R(d_{32}) \leq R(d_{23}) \leq R(d_{33}).$$

The utility function uses two weights, ω_1 and ω_2, with ω_1=0.33 and ω_2=1.09, as used by Barnett et al. [1].

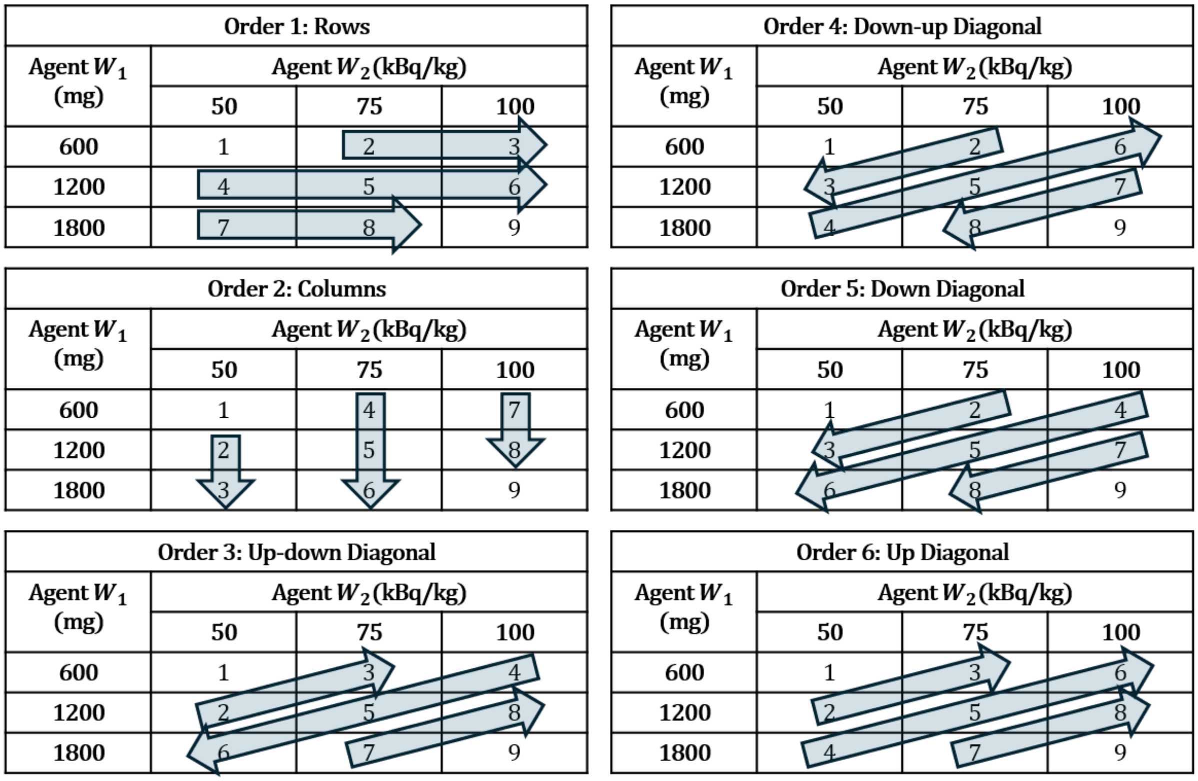

Figure 2: Visualisation of the six partial orderings considered in the 3×3 grid.

2.0.3 Joint TITE-BLRM

For each agent, the prior is elicited on the $\boldsymbol{\beta}$ in the same way as the Joint TITE-POCRM in Equation 1, with values calibrated via the procedure outline in Section 2.0.1. Values of these hyper-parameters for each of the agents are given in Table 1.

The interaction parameter between activity and toxicity is given a vague prior, $\psi \sim N(0, 100)$. The interaction parameter between the agents W_1 and W_2 for both activity and toxicity is given a half-normal prior $\eta^{(T)}, \eta^{(A)} \sim HN(0, 1)$.

The utility function uses the same weights as the Joint TITE-POCRM, ω_1=0.33 and ω_2=1.09.

2.0.4 TITE-BOIN12

The utility, u has a $Beta(1, 1)$ prior, with $\Psi_{Y^{(T)}Y^{(A)}}$ defined as: $\Psi_{01} = 100$, $\Psi_{00} = 40$, $\Psi_{10} = 0$, $\Psi_{11} = 60$, as suggested by Zhou et al [8].

3 Likelihoods

3.1 Joint TITE-POCRM

The G_{P2} for activity and toxicity are related by a Gumbel Model considering all binary combinations of toxicity and activity outcomes:

$$\begin{aligned}\pi_{y_\ell^{(A)},y_\ell^{(T)}}(x^{(A)}_{\ell,m_A^*}, x^{(T)}_{\ell,m_T^*}, \boldsymbol{\beta}, \boldsymbol{w_\ell}) =& (G_{P2}(x^{(A)}_{\ell,m_A^*}, w_\ell^{(A)}, \boldsymbol{\beta^{(A)}}))^{y_\ell^{(A)}}\\ & (1 - G_{P2}(x^{(A)}_{\ell,m_A^*}, w_\ell^{(A)}, \boldsymbol{\beta^{(A)}}))^{1-y_\ell^{(A)}}\\ & (G_{P2}(x^{(T)}_{\ell,m_T^*}, w_\ell^{(T)}, \boldsymbol{\beta^{(T)}}))^{y_\ell^{(T)}}\\ & (1 - G_{P2}(x^{(T)}_{\ell,m_T^*}, w_\ell^{(T)}, \boldsymbol{\beta^{(T)}}))^{1-y_\ell^{(T)}} +\\ & (-1)^{y_\ell^{(A)}+y_\ell^{(T)}}(G_{P2}(x^{(A)}_{\ell,m_A^*}, w_\ell^{(A)}, \boldsymbol{\beta^{(A)}}))\\ & (1 - G_{P2}(x^{(A)}_{\ell,m_A^*}, w_\ell^{(A)}, \boldsymbol{\beta^{(A)}}))\\ & G_{P2}(x^{(T)}_{\ell,m_T^*}, w_\ell^{(T)}, \boldsymbol{\beta^{(T)}}))\\ & (1 - G_{P2}(x^{(T)}_{\ell,m_T^*}, w_\ell^{(T)}, \boldsymbol{\beta^{(T)}}))\left(\frac{e^\psi - 1}{e^\psi + 1}\right),\end{aligned}$$

where $\pi_{y_\ell^{(A)},y_\ell^{(T)}}$ is the probability of observing each of the four combinations of binary activity outcomes, $\boldsymbol{\beta} = (\boldsymbol{\beta^{(A)}}, \boldsymbol{\beta^{(T)}})$, $\boldsymbol{w_\ell} = (w_\ell^{(A)}, w_\ell^{(T)})$ and $\psi \in (-\infty, \infty)$ is a correlation parameter of the distribution, with positive values of ψ corresponding to a positive correlation between activity and toxicity, negative values corresponding to a negative correlation, and larger absolute values of ψ corresponding to stronger correlations.

The likelihood is then:

$$\mathcal{L}_{P2}(\boldsymbol{\beta}) = \prod_{\ell=1}^{n}\prod_{a=0}^{1}\prod_{b=0}^{1}\{\pi_{a,b}(x^{(A)}_{\ell,m_A^*}, x^{(T)}_{\ell,m_T^*}, \boldsymbol{\beta}, \boldsymbol{w_\ell})\}^{\mathbb{I}((Y_\ell^{(A)},Y_\ell^{(T)})=(a,b))}, \tag{2}$$

for patients $\ell = 1, \ldots, n$.

3.2 Joint TITE-BLRM

The likelihood for the Joint TITE-BLRM is:

$$\mathcal{L}_{B}(\boldsymbol{\beta}, \boldsymbol{\eta}, \psi) = \prod_{\ell=1}^{n}\prod_{a=0}^{1}\prod_{b=0}^{1}\{\pi_{a,b}(x_\ell^{W_1}, x_\ell^{W_2}, \boldsymbol{\beta}, \boldsymbol{\eta}, \psi, \boldsymbol{w_\ell})\}^{\mathbb{I}((Y_\ell^{(A)},Y_\ell^{(T)})=(a,b))}, \tag{3}$$

where

$$\pi_{y_\ell^{(A)},y_\ell^{(T)}}(x_\ell^{W_1}, x_\ell^{W_2}, \boldsymbol{\beta}, \boldsymbol{\eta}, \psi, \boldsymbol{w_\ell}) =$$
$$(G_B(x_\ell^{W_1}, x_\ell^{W_2}, w_\ell^{(A)}, \boldsymbol{\beta^{(W_1,A)}}, \boldsymbol{\beta^{(W_2,A)}}, \eta^{(A)})^{y_\ell^{(A)}}$$
$$(1 - G_B(x_\ell^{W_1}, x_\ell^{W_2}, w_\ell^{(A)}, \boldsymbol{\beta^{(W_1,A)}}, \boldsymbol{\beta^{(W_2,A)}}, \eta^{(A)})^{1-y_\ell^{(A)}}$$
$$(G_B(x_\ell^{W_1}, x_\ell^{W_2}, w_\ell^{(T)}, \boldsymbol{\beta^{(W_1,T)}}, \boldsymbol{\beta^{(W_2,T)}}, \eta^{(T)})^{y_\ell^{(T)}}$$
$$(1 - G_B(x_\ell^{W_1}, x_\ell^{W_2}, w_\ell^{(T)}, \boldsymbol{\beta^{(W_1,T)}}, \boldsymbol{\beta^{(W_2,T)}}, \eta^{(T)})^{1-y_\ell^{(T)}} +$$
$$(-1)^{y_\ell^{(A)}+y_\ell^{(T)}}(G_B(x_\ell^{W_1}, x_\ell^{W_2}, w_\ell^{(A)}, \boldsymbol{\beta^{(W_1,A)}}, \boldsymbol{\beta^{(W_2,A)}}, \eta^{(A)})$$
$$(1 - G_B(x_\ell^{W_1}, x_\ell^{W_2}, w_\ell^{(A)}, \boldsymbol{\beta^{(W_1,A)}}, \boldsymbol{\beta^{(W_2,A)}}, \eta^{(A)})$$
$$G_B(x_\ell^{W_1}, x_\ell^{W_2}, w_\ell^{(T)}, \boldsymbol{\beta^{(W_1,T)}}, \boldsymbol{\beta^{(W_2,T)}}, \eta^{(T)})$$
$$(1 - G_B(x_\ell^{W_1}, x_\ell^{W_2}, w_\ell^{(T)}, \boldsymbol{\beta^{(W_1,T)}}, \boldsymbol{\beta^{(W_2,T)}}, \eta^{(T)})) \left(\frac{e^\psi - 1}{e^\psi + 1}\right),$$

where $\boldsymbol{\beta} = (\boldsymbol{\beta^{(W_1,A)}}, \boldsymbol{\beta^{(W_2,A)}}, \boldsymbol{\beta^{(W_1,T)}}, \boldsymbol{\beta^{(W_2,T)}})$ and $\boldsymbol{\eta} = (\eta^{(A)}, \eta^{(T)})$

4 Utility Scenarios

The utility for each dose combination in each of the 36 scenarios for each method is given in the following tables.

Acceptable dose combinations are highlighted in *italics*, good dose combinations are underlined and correct dose combinations are highlighted in **boldface**.

	Agent W_1 (mg)	A1					A2					A3				
		Agent W_2 (kBq/kg)														
		50	75	100	125	150	50	75	100	125	150	50	75	100	125	150
T1	600	*0.190*	*0.277*	*0.314*	*0.451*	*0.484*	*0.290*	*0.297*	*0.344*	*0.351*	***0.394***	0.050	0.057	0.084	*0.151*	*0.234*
	1200	*0.234*	*0.370*	*0.407*	*0.518*	***0.551***	*0.324*	*0.330*	*0.377*	*0.358*	*0.381*	0.084	0.120	*0.207*	*0.268*	***0.301***
T2	600	*0.167*	*0.251*	*0.284*	***0.401***	-0.672	*0.267*	*0.271*	***0.314***	*0.301*	-0.762	0.027	0.031	0.054	***0.101***	-0.922
	1200	-0.989	-0.855	-0.822	-0.688	-0.638	-0.899	-0.895	-0.852	-0.848	-0.808	-1.139	-1.105	-1.022	-0.938	-0.888
T3	600	*0.184*	*0.274*	*0.301*	***0.434***	-0.689	*0.284*	*0.294*	*0.331*	***0.334***	-0.779	0.044	0.054	0.071	*0.134*	-0.939
	1200	*0.217*	*0.360*	*0.351*	-0.622	-0.605	*0.307*	*0.320*	*0.321*	-0.782	-0.775	0.067	0.110	***0.151***	-0.872	-0.855
T4	600	*0.167*	***0.234***	-0.872	-0.755	-0.738	***0.267***	*0.254*	-0.842	-0.855	-0.828	0.027	0.014	-1.102	-1.055	-0.988
	1200	*0.151*	-0.839	-0.822	-0.688	-0.638	*0.241*	-0.879	-0.852	-0.848	-0.808	0.001	-1.089	-1.022	-0.938	-0.888
T5	600	***0.101***	-0.939	-0.905	-0.772	-0.738	***0.201***	-0.919	-0.875	-0.872	-0.828	-0.039	-1.159	-1.135	-1.072	-0.988
	1200	-0.972	-0.855	-0.822	-0.688	-0.638	-0.882	-0.895	-0.852	-0.848	-0.808	-1.122	-1.105	-1.022	-0.938	-0.888
T6	600	-1.022	-0.922	-0.905	-0.755	-0.738	-0.922	-0.902	-0.875	-0.855	-0.828	-1.162	-1.142	-1.135	-1.055	-0.988
	1200	-0.972	-0.822	-0.805	-0.655	-0.638	-0.882	-0.862	-0.835	-0.815	-0.808	-1.122	-1.072	-1.005	-0.905	-0.888

Table 2: Utility

	Agent W_1 (mg)	A4					A5					A6				
		Agent W_2 (kBq/kg)														
		50	75	100	125	150	50	75	100	125	150	50	75	100	125	150
T1	600	0.040	*0.177*	*0.264*	*0.351*	*0.434*	0.090	0.097	0.104	0.111	0.114	0.090	0.077	0.064	0.051	0.034
	1200	0.084	*0.220*	*0.307*	*0.368*	***0.451***	*0.184*	*0.270*	*0.357*	*0.418*	***0.501***	0.084	0.070	0.057	0.018	***0.101***
T2	600	0.017	*0.151*	*0.234*	***0.301***	-0.722	0.067	0.071	0.074	0.061	-1.042	0.067	0.051	0.034	0.001	-1.122
	1200	-1.139	-1.005	-0.922	-0.838	-0.738	-1.039	-0.955	-0.872	-0.788	-0.688	-1.139	-1.155	-1.172	-1.188	-1.088
T3	600	0.034	*0.174*	*0.251*	***0.334***	-0.739	0.084	0.094	0.091	0.094	-1.059	0.084	0.074	0.051	0.034	-1.139
	1200	0.067	*0.210*	*0.251*	-0.772	-0.705	*0.167*	*0.260*	***0.301***	-0.722	-0.655	0.067	0.060	0.001	-1.122	-1.055
T4	600	0.017	***0.134***	-0.922	-0.855	-0.788	0.067	0.054	-1.082	-1.095	-1.108	0.067	0.034	-1.122	-1.155	-1.188
	1200	0.001	-0.989	-0.922	-0.838	-0.738	***0.101***	-0.939	-0.872	-0.788	-0.688	0.001	-1.139	-1.172	-1.188	-1.088
T5	600	-0.049	-1.039	-0.955	-0.872	-0.788	0.001	-1.119	-1.115	-1.112	-1.108	0.001	-1.139	-1.155	-1.172	-1.188
	1200	-1.122	-1.005	-0.922	-0.838	-0.738	-1.022	-0.955	-0.872	-0.788	-0.688	-1.122	-1.155	-1.172	-1.188	-1.088
T6	600	-1.172	-1.022	-0.955	-0.855	-0.788	-1.122	-1.102	-1.115	-1.095	-1.108	-1.122	-1.122	-1.155	-1.155	-1.188
	1200	-1.122	-0.972	-0.905	-0.805	-0.738	-1.022	-0.922	-0.855	-0.755	-0.688	-1.122	-1.122	-1.155	-1.155	-1.088

Table 3: Utility

	Agent W_1 (mg)	A7 50	75	100	125	150	A8 50	75	100	125	150
		Agent W_2 (kBq/kg)									
T1	600	*0.1901*	*0.2769*	*0.3637*	*0.3505*	*0.334*	*0.1901*	*0.2769*	*0.3637*	*0.2505*	*0.134*
	1200	*0.2335*	*0.3203*	***0.4071***	*0.3675*	*0.351*	*0.2835*	*0.3203*	***0.4071***	*0.2675*	*0.151*
T2	600	*0.167*	*0.2505*	***0.334***	*0.301*	-0.822	*0.167*	*0.2505*	***0.334***	*0.201*	-1.022
	1200	-0.9885	-0.905	-0.8215	-0.838	-0.838	-0.9385	-0.905	-0.8215	-0.938	-1.038
T3	600	*0.1835*	*0.2736*	*0.3505*	*0.334*	-0.8385	*0.1835*	*0.2736*	*0.3505*	*0.234*	-1.0385
	1200	*0.217*	*0.3104*	***0.351***	-0.772	-0.805	*0.267*	*0.3104*	***0.351***	-0.872	-1.005
T4	600	*0.167*	***0.234***	-0.822	-0.855	-0.888	*0.167*	***0.234***	-0.822	-0.955	-1.088
	1200	*0.151*	-0.8885	-0.8215	-0.838	-0.838	*0.201*	-0.8885	-0.8215	-0.938	-1.038
T5	600	***0.101***	-0.9385	-0.855	-0.8715	-0.888	***0.101***	-0.9385	-0.855	-0.9715	-1.088
	1200	-0.972	-0.905	-0.8215	-0.838	-0.838	-0.922	-0.905	-0.8215	-0.938	-1.038
T6	600	-1.022	-0.922	-0.855	-0.855	-0.888	-1.022	-0.922	-0.855	-0.955	-1.088
	1200	-0.972	-0.872	-0.805	-0.805	-0.838	-0.922	-0.872	-0.805	-0.905	-1.038

Table 4: Utility

	Agent W_1 (mg)	A1 50	75	100	125	150	A2 50	75	100	125	150	A3 50	75	100	125	150
		Agent W_2 (kBq/kg)														
T1	600	*0.1967*	*0.2923*	*0.3379*	*0.4835*	*0.528*	*0.2967*	*0.3123*	*0.3679*	*0.3835*	*0.438*	0.0567	0.0723	0.1079	*0.1835*	*0.278*
	1200	*0.2445*	*0.3901*	*0.4357*	*0.5725*	***0.617***	*0.3345*	*0.3501*	***0.4057***	*0.4125*	***0.447***	0.0945	0.1401	*0.2357*	***0.3225***	***0.367***
T2	600	*0.189*	*0.2835*	*0.328*	***0.467***	0.506	*0.289*	*0.3035*	*0.358*	***0.367***	0.416	0.049	0.0635	0.098	***0.167***	0.256
	1200	0.2005	0.345	0.3895	0.534	0.584	0.2905	0.305	0.3595	0.374	0.414	0.0505	0.095	0.1895	0.284	0.334
T3	600	*0.1945*	*0.2912*	*0.3335*	***0.478***	0.5005	*0.2945*	*0.3112*	*0.3635*	*0.378*	0.4105	0.0545	0.0712	0.1035	*0.178*	0.2505
	1200	*0.239*	*0.3868*	*0.417*	0.556	0.595	*0.329*	*0.3468*	***0.387***	0.396	0.425	0.089	0.1368	***0.217***	0.306	0.345
T4	600	*0.189*	***0.278***	0.306	0.445	0.484	*0.289*	*0.298*	0.336	0.345	0.394	0.049	0.058	0.076	0.145	0.234
	1200	0.217	0.3505	0.3895	0.534	0.584	***0.307***	0.3105	0.3595	0.374	0.414	0.067	0.1005	0.1895	0.284	0.334
T5	600	***0.167***	0.2505	0.295	0.4395	0.484	***0.267***	0.2705	0.325	0.3395	0.394	0.027	0.0305	0.065	0.1395	0.234
	1200	0.206	0.345	0.3895	0.534	0.584	0.296	0.305	0.3595	0.374	0.414	0.056	0.095	0.1895	0.284	0.334
T6	600	0.156	0.256	0.295	0.445	0.484	0.256	0.276	0.325	0.345	0.394	0.016	0.036	0.065	0.145	0.234
	1200	0.206	0.356	0.395	0.545	0.584	0.296	0.316	0.365	0.385	0.414	0.056	0.106	0.195	0.295	0.334

Table 5: Alternative BOIN Utility

		A4					A5					A6				
	Agent W_1	Agent W_2 (kBq/kg)														
	(mg)	50	75	100	125	150	50	75	100	125	150	50	75	100	125	150
T1	600	0.0467	*0.1923*	*0.2879*	*0.3835*	***0.478***	0.0967	0.1123	0.1279	0.1435	0.158	0.0967	0.0923	0.0879	0.0835	0.078
	1200	0.0945	*0.2401*	*0.3357*	*0.4225*	***0.517***	*0.1945*	*0.2901*	*0.3857*	*0.4725*	***0.567***	0.0945	0.0901	0.0857	0.0725	***0.167***
T2	600	0.039	*0.1835*	*0.278*	**0.367**	0.456	0.089	0.1035	0.118	0.127	0.136	0.089	0.0835	0.078	0.067	0.056
	1200	0.0505	0.195	0.2895	0.384	0.484	0.1505	0.245	0.3395	0.434	0.534	0.0505	0.045	0.0395	0.034	0.134
T3	600	0.0445	*0.1912*	*0.2835*	***0.378***	0.4505	0.0945	0.1112	0.1235	0.138	0.1305	0.0945	0.0912	0.0835	0.078	0.0505
	1200	0.089	*0.2368*	*0.317*	0.406	0.495	*0.189*	*0.2868*	***0.367***	0.456	0.545	0.089	0.0868	0.067	0.056	0.145
T4	600	0.039	***0.178***	0.256	0.345	0.434	0.089	0.098	0.096	0.105	0.114	0.089	0.078	0.056	0.045	0.034
	1200	0.067	0.2005	0.2895	0.384	0.484	***0.167***	0.2505	0.3395	0.434	0.534	0.067	0.0505	0.0395	0.034	0.134
T5	600	0.017	0.1505	0.245	0.3395	0.434	0.067	0.0705	0.085	0.0995	0.114	0.067	0.0505	0.045	0.0395	0.034
	1200	0.056	0.195	0.2895	0.384	0.484	0.156	0.245	0.3395	0.434	0.534	0.056	0.045	0.0395	0.034	0.134
T6	600	0.006	0.156	0.245	0.345	0.434	0.056	0.076	0.085	0.105	0.114	0.056	0.056	0.045	0.045	0.034
	1200	0.056	0.206	0.295	0.395	0.484	0.156	0.256	0.345	0.445	0.534	0.056	0.056	0.045	0.045	0.134

Table 6: Alternative BOIN Utility

		A7					A8				
	Agent W_1	Agent W_2 (kBq/kg)									
	(mg)	50	75	100	125	150	50	75	100	125	150
T1	600	*0.1967*	*0.2923*	*0.3879*	*0.3835*	*0.378*	*0.1967*	*0.2923*	*0.3879*	*0.2835*	*0.178*
	1200	*0.2445*	*0.3401*	***0.4357***	*0.4225*	*0.417*	*0.2945*	*0.3401*	***0.4357***	*0.3225*	*0.217*
T2	600	*0.189*	*0.2835*	***0.378***	*0.367*	0.356	*0.189*	*0.2835*	***0.378***	*0.267*	0.156
	1200	0.2005	0.295	0.3895	0.384	0.384	0.2505	0.295	0.3895	0.284	0.184
T3	600	*0.1945*	*0.2912*	*0.3835*	*0.378*	0.3505	*0.1945*	*0.2912*	*0.3835*	*0.278*	0.1505
	1200	*0.239*	*0.3368*	***0.417***	0.406	0.395	*0.289*	*0.3368*	***0.417***	0.306	0.195
T4	600	*0.189*	***0.278***	0.356	0.345	0.334	*0.189*	***0.278***	0.356	0.245	0.134
	1200	*0.217*	0.3005	0.3895	0.384	0.384	*0.267*	0.3005	0.3895	0.284	0.184
T5	600	***0.167***	0.2505	0.345	0.3395	0.334	***0.167***	0.2505	0.345	0.2395	0.134
	1200	0.206	0.295	0.3895	0.384	0.384	0.256	0.295	0.3895	0.284	0.184
T6	600	0.156	0.256	0.345	0.345	0.334	0.156	0.256	0.345	0.245	0.134
	1200	0.206	0.306	0.395	0.395	0.384	0.256	0.306	0.395	0.295	0.184

Table 7: Alternative BOIN Utility

5 Non-parametric Benchmark

In order to give the results for each of the scenarios a relevant interpretation, they are compared to the non-parametric benchmark for unknown orderings [5].

For this work, we have also made the modification to this benchmark to restrict selections based on admissibility, so that the comparison of results are meaningful. For exact details of the benchmark for unknown ordering, we refer the reader to the original manuscript by Mozgunov et al [5]. In summary, like the traditional non-parametric benchmark, the complete information for all patients (the maximum sample size) on all doses is considered in order to find the dose that is closest to the target for some criterion. In the case of unknown ordering, this benchmark assesses the likelihood of each ordering, given the complete information on each patient. Since in the implementation of the Joint TITE-POCRM and Joint TITE-BLRM, the admissibility of each dose combination is an important feature in the recommendation of dose combination, this should also be included in the benchmark. Therefore, the same admissibility criteria that is applied to the dose combinations for each of the applied methods based on their models is applied to the complete information. In this way, the benchmark is also able to recommend ”No admissible dose” when none of the doses are admissible based on the complete information.

6 Results for Alternative BOIN Utility

Here we present the equivalent figures for figures 2-9 in the main manuscript, but the Joint TITE-BOIN design uses the alternative BOIN Utility.

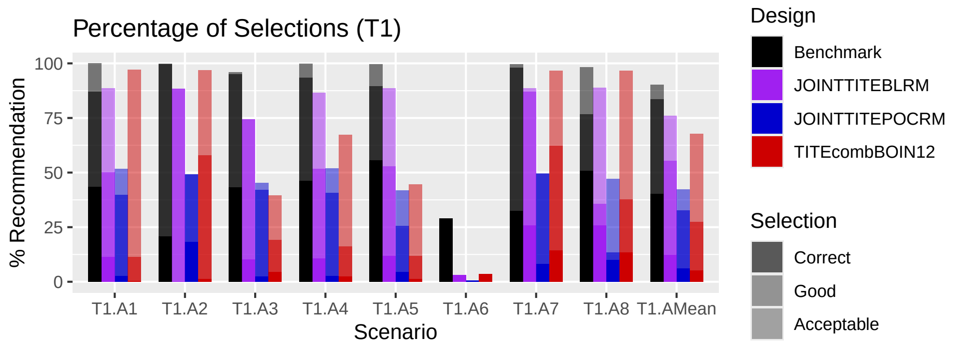

(a) Percentage of Correct, Good and Acceptable selections

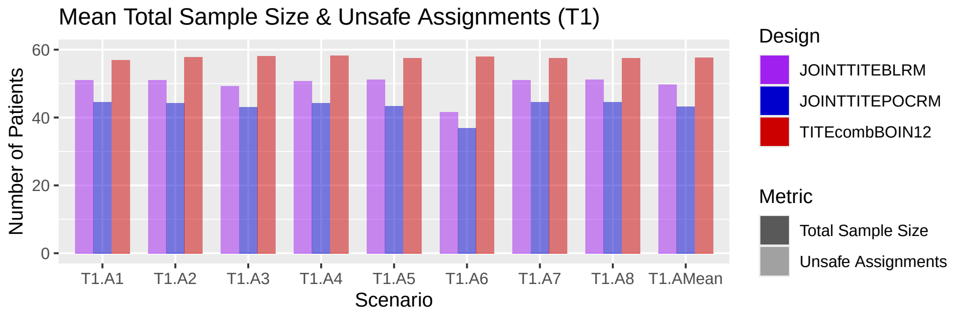

(b) Mean total patients, and mean patients assigned to unsafe dose combinations.

Figure 3: Results for scenarios T1.A1 - T1.A6, including an average over these six scenarios, labelled T1.AMean. (Alternative BOIN Utility)

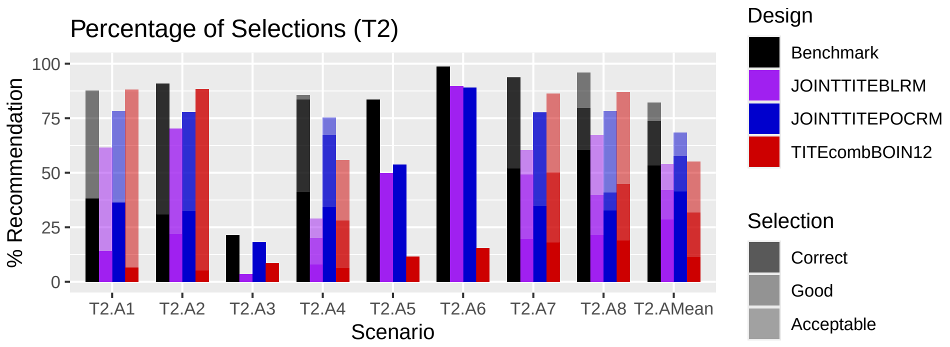

(a) Percentage of Correct, Good and Acceptable selections

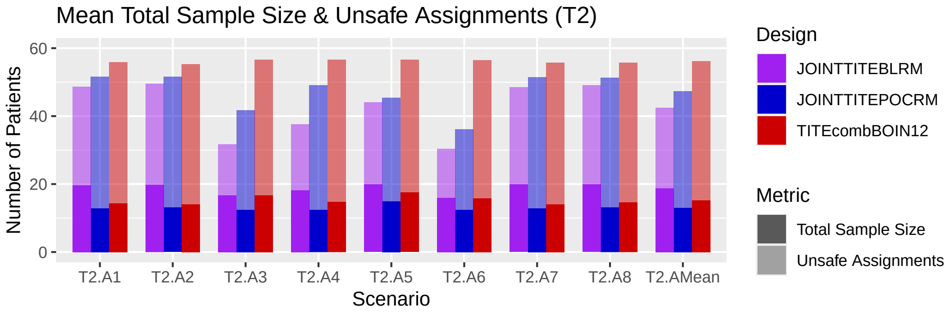

(b) Mean total patients, and mean patients assigned to unsafe dose combinations.

Figure 4: Results for scenarios T2.A1 - T2.A6, including an average over these six scenarios, labelled T2.AMean. (Alternative BOIN Utility)

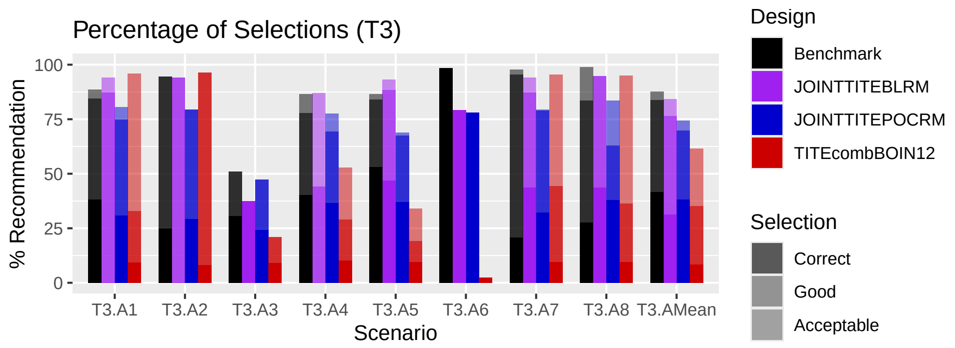

(a) Percentage of Correct, Good and Acceptable selections

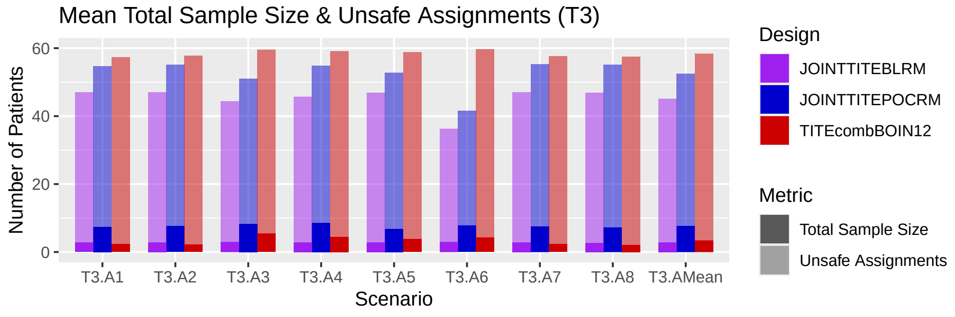

(b) Mean total patients, and mean patients assigned to unsafe dose combinations.

Figure 5: Results for scenarios T3.A1 - T3.A6, including an average over these six scenarios, labelled T3.AMean. (Alternative BOIN Utility)

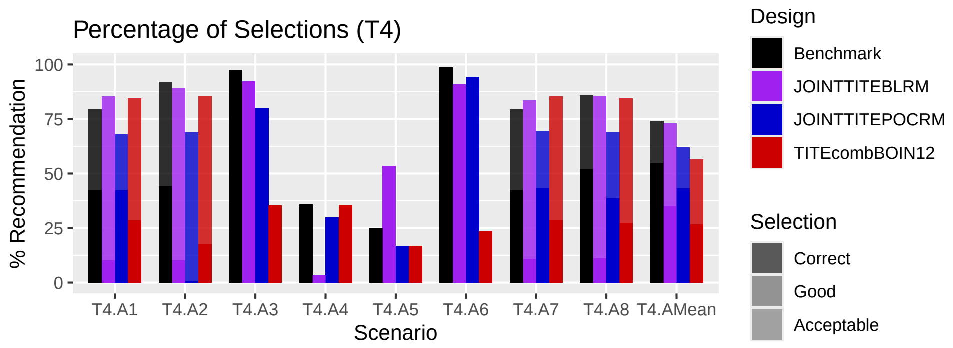

(a) Percentage of Correct, Good and Acceptable selections

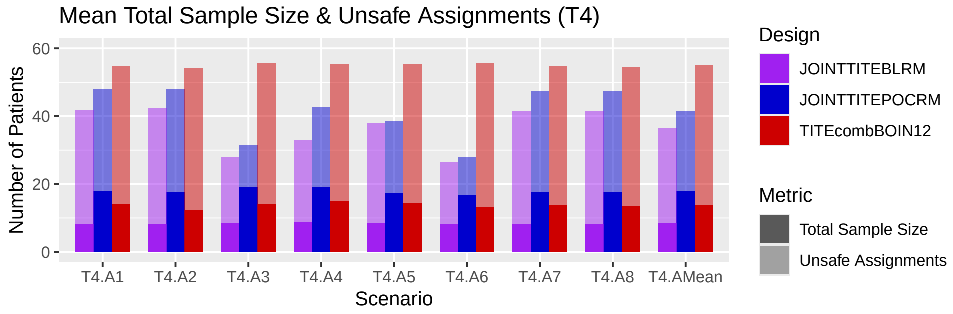

(b) Mean total patients, and mean patients assigned to unsafe dose combinations.

Figure 6: Results for scenarios T4.A1 - T4.A6, including an average over these six scenarios, labelled T4.AMean. (Alternative BOIN Utility)

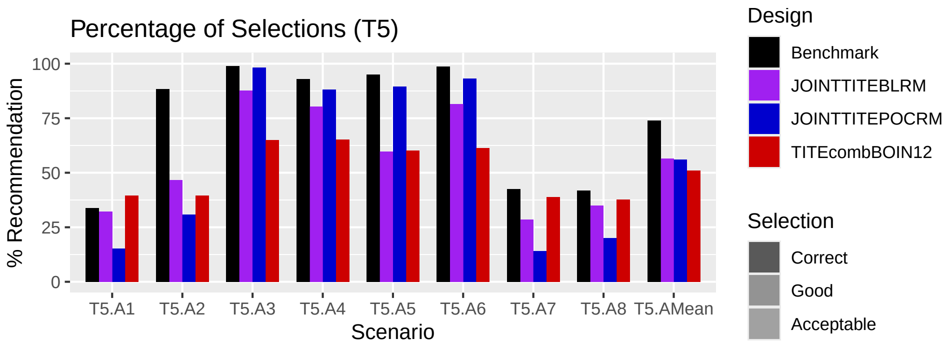

(a) Percentage of Correct, Good and Acceptable selections

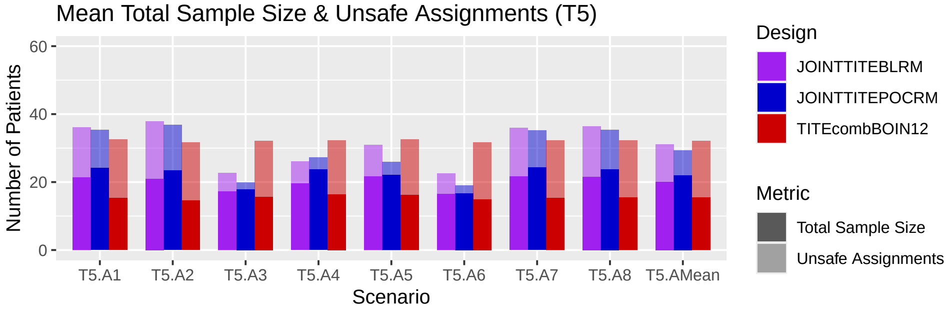

(b) Mean total patients, and mean patients assigned to unsafe dose combinations.

Figure 7: Results for scenarios T5.A1 - T5.A6, including an average over these six scenarios, labelled T5.AMean. (Alternative BOIN Utility)

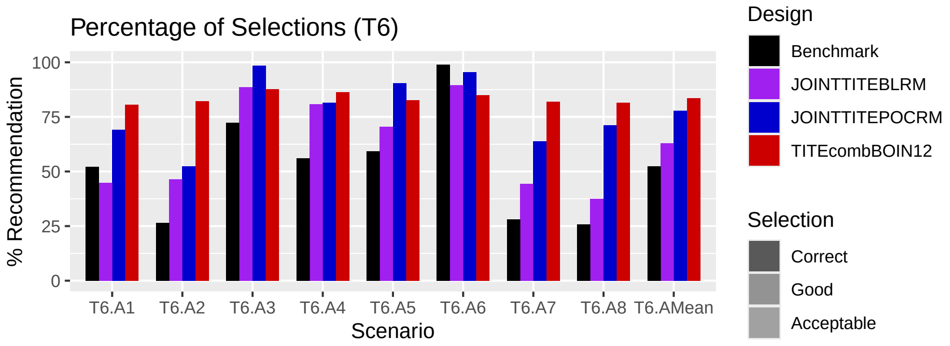

(a) Percentage of Correct, Good and Acceptable selections

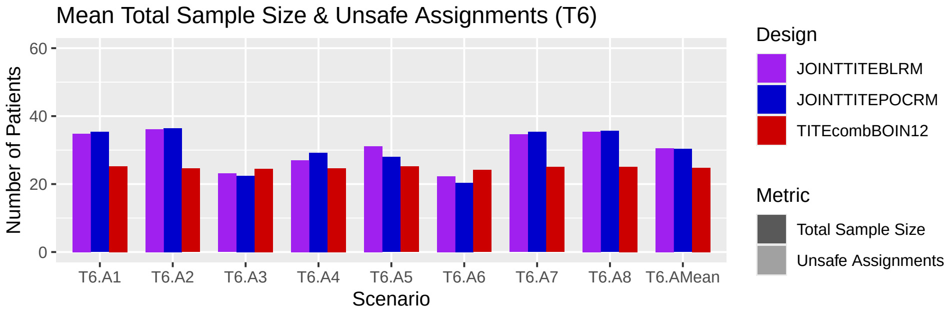

(b) Mean total patients, and mean patients assigned to unsafe dose combinations.

Figure 8: Results for scenarios T6.A1 - T6.A6, including an average over these six scenarios, labelled T6.AMean. (Alternative BOIN Utility)

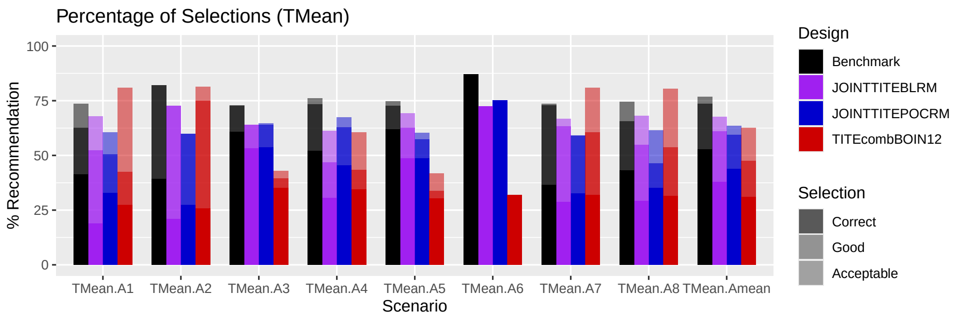

(a) Percentage of Correct, Good and Acceptable selections

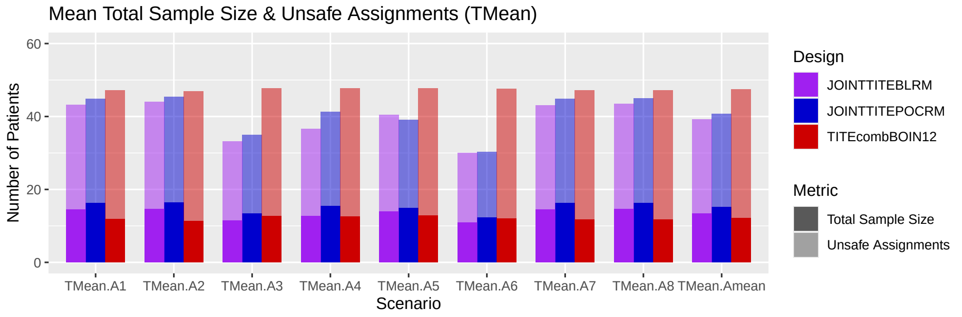

(b) Mean total patients, and mean patients assigned to unsafe dose combinations. (Alternative BOIN Utility)

Figure 9: Average results over toxicity scenarios, labelled TMean.A1 - TMean.A6, including an average over all 36 scenarios, labelled TMean.AMean. (Alternative BOIN Utility)

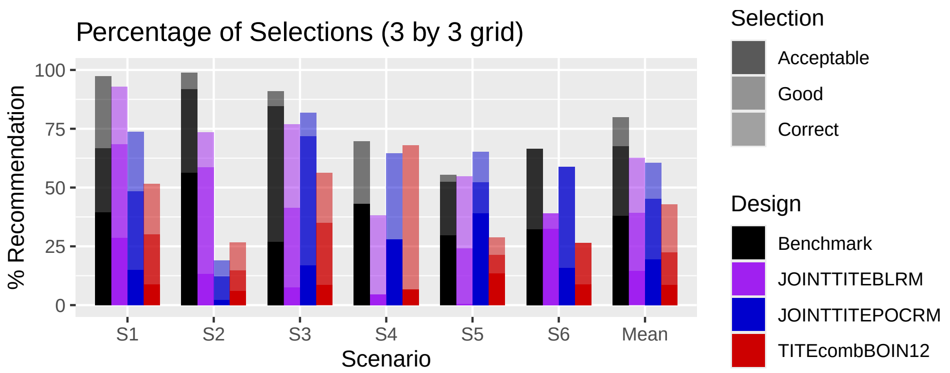

(a) Percentage of Correct, Good and Acceptable selections

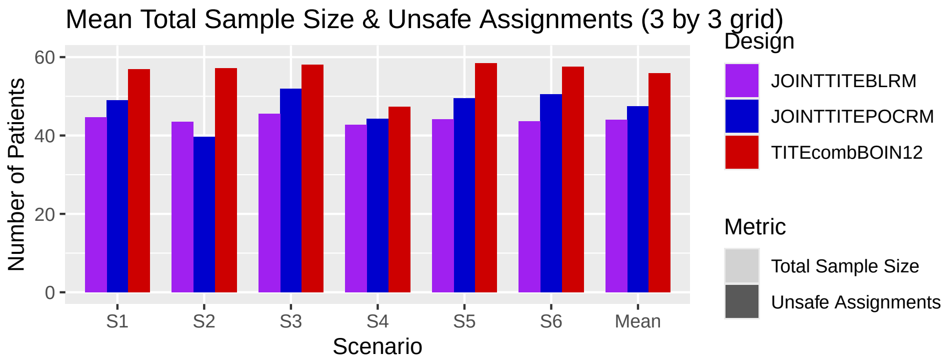

(b) Mean total patients, and mean patients assigned to unsafe dose combinations.

Figure 10: Results for the scenarios with 3 by 3 grids, including an average over these scenarios. (Alternative BOIN Utility)

7 All Assignments

Acceptable dose combinations are highlighted in *italics*, good dose combinations are <u>underlined</u> and correct dose combinations are highlighted in **boldface**.

	Agent W_1 (mg)	A1 Agent W_2 (KBq/kg)					A2					A3				
		50	75	100	125	150	50	75	100	125	150	50	75	100	125	150
T1	600	*0.7*	*0*	*1.5*	<u>*0.1*</u>	<u>*0*</u>	<u>*0.7*</u>	<u>*0*</u>	<u>*1.6*</u>	<u>*0.1*</u>	<u>*0*</u>	0.7	0	2	<u>*0.4*</u>	<u>*0*</u>
	1200	*3.3*	*12.5*	*15.4*	<u>*13.8*</u>	<u>***3.8***</u>	*3.3*	<u>*12.3*</u>	<u>***15.4***</u>	<u>*13.9*</u>	<u>*3.9*</u>	2.3	11.1	<u>*15.3*</u>	<u>*13.6*</u>	**3.8**
T2	600	*9.2*	*9.6*	<u>7.2</u>	<u>***2.8***</u>	0.5	<u>*10.7*</u>	<u>*9.4*</u>	<u>***7***</u>	<u>*2.8*</u>	0.5	4.5	3.3	4.7	<u>***2.4***</u>	0.4
	1200	14.2	4.8	0.2	0	0	14.2	4.8	0.2	0	0	10.6	5.4	0.3	0	0
T3	600	*1*	*0*	1.4	<u>***0***</u>	0	<u>*1*</u>	<u>*0*</u>	<u>***1.5***</u>	<u>*0*</u>	0	0.9	0	1.9	<u>***0.2***</u>	0
	1200	*5.4*	<u>*20*</u>	16.3	2.5	0.3	<u>*5.4*</u>	<u>*20.1*</u>	<u>*16.3*</u>	2.6	0.3	3.5	18	<u>*16.9*</u>	2.7	0.2
T4	600	*6.6*	<u>***4***</u>	1.6	0.1	0	<u>***7.2***</u>	<u>*4.1*</u>	1.7	0.1	0	3.8	1.3	1.3	0.2	0
	1200	22.9	6.3	0.2	0	0	*22.9*	6.3	0.2	0	0	14.3	6.8	0.2	0	0
T5	600	<u>***14.8***</u>	2.1	0.8	0	0	<u>***16.8***</u>	1.9	0.8	0	0	5.5	0.9	0.9	0.1	0
	1200	14.1	4.3	0	0	0	14	4.3	0.1	0	0	10.6	4.6	0.1	0	0
T6	600	11.8	1.9	1	0	0	13.2	1.8	1	0	0	4.4	1	1.1	0.1	0
	1200	14.5	5.4	0.2	0	0	14.5	5.4	0.2	0	0	10.3	6	0.2	0	0

Table 8: Joint TITE-BLRM Assignments 1

	Agent W_1 (mg)	A4 Agent W_2 (KBq/kg)					A5					A6				
		50	75	100	125	150	50	75	100	125	150	50	75	100	125	150
T1	600	0.7	*0*	*1.8*	*0.2*	<u>*0*</u>	0.7	0	1.7	0.2	0	0.7	0	2.2	0.6	0.1
	1200	2.6	*12.1*	*15.5*	<u>*14.1*</u>	<u>***3.7***</u>	*3*	*12.1*	*15.4*	<u>*14.3*</u>	<u>***3.9***</u>	2.2	9.9	12.4	10.3	<u>***3.4***</u>
T2	600	4.9	*6*	<u>*6.3*</u>	<u>***2.3***</u>	0.4	6.4	7.7	7.2	3	0.5	5	3.7	3.9	1.8	0.3
	1200	12.4	5.1	0.2	0	0	14.1	5	0.2	0	0	10.1	5.2	0.4	0	0
T3	600	0.9	*0*	<u>*1.7*</u>	<u>***0.1***</u>	0	0.9	0	1.6	0.1	0	0.9	0	2	0.3	0
	1200	4	*19.8*	<u>*16.3*</u>	2.6	0.2	*4.8*	<u>*19.8*</u>	<u>***16.9***</u>	2.7	0.2	3.3	14.4	12.4	2.6	0.3
T4	600	4.1	<u>***2.5***</u>	1.6	0.1	0	4.7	3.1	1.7	0.2	0	3.9	1.6	1.2	0.2	0
	1200	17.5	6.7	0.2	0	0	<u>***21.7***</u>	6.4	0.2	0	0	12.9	6.4	0.3	0	0
T5	600	6.5	1.5	0.9	0	0	9.4	2.2	0.8	0	0	6.2	0.9	0.8	0.1	0
	1200	12.6	4.4	0.1	0	0	14.2	4.3	0.1	0	0	10.1	4.4	0.1	0	0
T6	600	5.4	1.7	1.2	0.1	0	7.8	2.1	1.1	0	0	4.8	0.9	1.2	0.1	0
	1200	12.6	5.8	0.2	0	0	14.1	5.7	0.2	0	0	9.5	5.6	0.3	0	0

Table 9: Joint TITE-BLRM Assignments 2

	Agent W_1 (mg)	A7 Agent W_2 (kBq/kg)					A8				
		50	75	100	125	150	50	75	100	125	150
T1	600	*0.7*	*0*	*1.6*	*0.1*	*0*	*0.7*	*0*	*1.6*	*0.1*	*0*
	1200	*3.2*	*12.4*	***15.2***	*14.1*	*3.9*	*3.3*	*12.4*	***15.4***	*14.1*	*3.8*
T2	600	*9.2*	*9.6*	***7.1***	*2.8*	0.5	*9.3*	*9.7*	***7.3***	*2.9*	0.5
	1200	14.3	4.9	0.2	0	0	14.3	4.9	0.2	0	0
T3	600	*1*	*0*	*1.5*	*0*	0	*1*	*0*	*1.5*	*0*	0
	1200	*5.3*	*19.9*	***16.4***	2.6	0.2	*5.3*	*20*	***16.3***	2.5	0.2
T4	600	*6.5*	***4.2***	1.7	0.1	0	*6.5*	***3.9***	1.7	0.1	0
	1200	*22.4*	6.4	0.2	0	0	*22.8*	6.3	0.2	0	0
T5	600	***14.3***	2.1	0.8	0	0	***14.8***	2.1	0.8	0	0
	1200	14.3	4.3	0.1	0	0	14.3	4.3	0.1	0	0
T6	600	11.4	2	1	0	0	12	2	1	0	0
	1200	14.6	5.4	0.2	0	0	14.7	5.4	0.2	0	0

Table 10: Joint TITE-BLRM Assignments 3

	Agent W_1 (mg)	A1 Agent W_2 (KBq/kg)					A2					A3				
		50	75	100	125	150	50	75	100	125	150	50	75	100	125	150
T1	600	*0.2*	*1*	*4.1*	*4.8*	*7.7*	*0.2*	*1*	*4.3*	*4.6*	*7.9*	0.1	1	4.3	*4.9*	*8*
	1200	*2.2*	*6.8*	*7.4*	*7.9*	***2.4***	*2.2*	*6.7*	***7.6***	*7.3*	*2.4*	1.2	6.6	*7.4*	*7.1*	**2.4**
T2	600	*2.5*	*8.3*	16	***12***	3.8	*2.5*	*8.1*	***15.7***	*12.1*	3.7	1.5	3.7	12.1	***12***	4
	1200	3.9	4.8	0.4	0	0	4.1	4.8	0.4	0.1	0	2.7	4.9	0.7	0.1	0
T3	600	*0.3*	*1.7*	7.1	***12.4***	3.7	*0.2*	*1.6*	***7.7***	*12.3*	4.1	0.2	1.3	6.6	***12.1***	4.5
	1200	*2.6*	*11.6*	11.5	3.3	0.4	*2.7*	*11.2*	*11.7*	3.2	0.4	1.6	10.2	*10.7*	3.3	0.5
T4	600	*3.1*	***16.5***	10	1.7	0.4	***3.4***	*15.6*	9.8	1.7	0.3	1.5	5.5	9.9	2.1	0.3
	1200	10.2	5.7	0.2	0	0	*11.5*	5.6	0.2	0	0	5.5	6.4	0.2	0	0
T5	600	***11.2***	8.4	3.8	0.4	0	***13.4***	8	3.8	0.4	0	2	4.5	4.6	0.6	0
	1200	7.8	3.6	0	0	0	7.6	3.6	0.1	0	0	4	4	0.1	0	0
T6	600	8.7	9	4.2	0.7	0.1	10.4	8.6	4.3	0.8	0.1	1.5	4.5	5.2	0.9	0.1
	1200	8	4.4	0.1	0	0	7.7	4.4	0.1	0	0	4.5	5.4	0.3	0	0

Table 11: Joint TITE-POCRM Assignments 1

	Agent W_1 (mg)	A4 50	A4 75	A4 100	A4 125	A4 150	A5 50	A5 75	A5 100	A5 125	A5 150	A6 50	A6 75	A6 100	A6 125	A6 150
		Agent W_2 (KBq/kg)														
T1	600	0.2	*1*	*4.9*	*5.8*	*9.2*	0.2	1	3.6	3	4	0.1	1.1	4.3	4.4	6.2
	1200	1.4	*6.1*	*6.5*	*6.8*	***2.4***	*2.2*	*7.9*	*9.6*	*9.4*	***2.7***	1.1	6.4	6	5.2	***2.1***
T2	600	1.7	*7.1*	*15.9*	***12.1***	4.1	1.9	4.7	11.6	12.2	4.6	1.5	3.6	9.7	8.9	4.2
	1200	2.8	4.9	0.5	0.1	0	4.9	5	0.4	0	0	2.8	4.7	0.6	0.1	0
T3	600	0.2	*1.7*	*8.5*	***14.5***	4.4	0.2	1.4	5.2	7.4	2.8	0.1	1.4	5.9	8.9	3.8
	1200	1.7	*9.2*	*10.4*	3.7	0.5	*3.1*	*15.6*	***13.1***	3.5	0.5	1.6	8.6	7.2	3.5	0.5
T4	600	1.7	***14.5***	10.5	1.7	0.3	1.9	7.9	8.9	2.2	0.4	1.4	5.3	8.5	2.1	0.3
	1200	7.4	6.2	0.2	0	0	***11.6***	5.7	0.2	0	0	4.3	5.7	0.3	0	0
T5	600	3.5	8.9	4.1	0.3	0.1	3.9	5.8	4.1	0.6	0.1	2.3	4.3	4.3	0.6	0.1
	1200	6.4	3.7	0.1	0	0	7.6	3.7	0.1	0	0	3.5	3.9	0.1	0	0
T6	600	2.8	9.6	4.9	0.6	0.1	2.8	6.1	4.6	1	0.2	1.3	4.2	4.8	1	0.1
	1200	6.3	4.7	0.2	0	0	8.3	4.8	0.2	0	0	3.7	4.9	0.3	0	0

Table 12: Joint TITE-POCRM Assignments 2

	Agent W_1 (mg)	A7 50	A7 75	A7 100	A7 125	A7 150	A8 50	A8 75	A8 100	A8 125	A8 150
		Agent W_2 (kBq/kg)									
T1	600	*0.2*	*1*	*4.5*	*4.7*	*7.8*	*0.2*	*1*	*4.4*	*4*	*5.2*
	1200	*2.1*	*6.7*	***7.6***	*7.6*	*2.5*	*2.4*	*7.1*	***9.1***	*8.5*	*2.6*
T2	600	*2.5*	*8*	***16.2***	*11.9*	3.9	*2.4*	*8*	***15.9***	*12*	3.8
	1200	3.8	4.7	0.4	0	0	4	4.9	0.4	0	0
T3	600	*0.3*	*1.6*	*8*	*12.4*	4	*0.3*	*1.6*	*7.7*	*10.3*	3.4
	1200	*2.6*	*11*	***12***	3.1	0.4	*2.8*	*12.2*	***13.2***	3.3	0.4
T4	600	*3*	***16.5***	10	1.7	0.3	*3.1*	***15.5***	9.7	1.8	0.4
	1200	*10.1*	5.6	0.2	0	0	*11.3*	5.5	0.2	0	0
T5	600	***10.9***	8.5	3.7	0.4	0	***11.6***	8.1	3.8	0.4	0
	1200	8	3.6	0.1	0	0	7.8	3.5	0.1	0	0
T6	600	8.5	9.3	4.3	0.7	0.1	9.1	8.8	4.4	0.7	0.1
	1200	7.8	4.4	0.1	0	0	8	4.3	0.1	0	0

Table 13: Joint TITE-POCRM Assignments 3

	Agent W_1 (mg)	A1 50	A1 75	A1 100	A1 125	A1 150	A2 50	A2 75	A2 100	A2 125	A2 150	A3 50	A3 75	A3 100	A3 125	A3 150
		Agent W_2 (KBq/kg)														
T1	600	*2*	*4.6*	*6.6*	*0.4*	*0*	*2.4*	*4.8*	*8.1*	*0.4*	*0*	3.6	5.7	8.2	*2.8*	*0.3*
	1200	*2.6*	*18.7*	*2.4*	*0*	***0***	*2.9*	*16.5*	***2.5***	*0*	*0*	5.3	13.1	*6.8*	*1*	**0**
T2	600	*16.5*	*14.6*	2.3	***0.2***	0	*18.1*	*12.1*	***2.3***	*0.2*	0	15.8	14.2	9.1	***1.8***	0.1
	1200	3.5	4.8	0.1	0	0	3.2	4.5	0.1	0	0	5.7	4.7	0.9	0.1	0
T3	600	*3.1*	*6*	6.1	***0.2***	0	*3.7*	*6.2*	***7.5***	*0.2*	0	5.4	7.8	8.8	***2.3***	0.1
	1200	*3.3*	*17.7*	1.1	0	0	*3.6*	*15.4*	*1.2*	0	0	6.5	14.1	*3.7*	0.3	0
T4	600	*16.7*	***12.8***	1.4	0	0	***18.2***	*11*	1.4	0	0	16.8	13.9	4.3	0.4	0
	1200	6	5.6	0.1	0	0	*5.9*	5.1	0.1	0	0	10.1	5.7	0.4	0	0
T5	600	***18.1***	3.9	0.6	0	0	***18.3***	3.6	0.6	0	0	17.5	4.8	0.9	0	0
	1200	3.7	4.3	0	0	0	3.4	4.2	0.1	0	0	5.1	3.9	0.1	0	0
T6	600	11.2	4.4	0.9	0	0	11.3	4.3	1	0	0	11.2	4.3	1.4	0.1	0
	1200	2.8	5.6	0.1	0	0	2.7	5.2	0.1	0	0	3.4	4.6	0.2	0	0

Table 14: TITE-comb-BOIN12 Assignments 1

	Agent W_1 (mg)	A4 50	A4 75	A4 100	A4 125	A4 150	A5 50	A5 75	A5 100	A5 125	A5 150	A6 50	A6 75	A6 100	A6 125	A6 150
		Agent W_2 (KBq/kg)														
T1	600	1.7	*6.1*	*9.8*	*1.1*	*0*	2.2	3.8	5.2	0.9	0.1	5.7	7.8	8.9	3.5	0.9
	1200	2.9	*14.6*	*4.1*	*0.1*	***0***	*4.4*	*18.4*	*4.6*	*0.4*	***0***	6.2	11	5.2	1.2	***0.3***
T2	600	11	*20.1*	*6.2*	***0.4***	0	16.1	15.5	6.1	1	0.1	17.7	14	6.6	1.3	0.1
	1200	4.3	4.5	0.2	0	0	5.3	5.1	0.6	0.1	0	5.1	4.2	0.8	0.1	0
T3	600	2.6	*8.5*	*9.5*	***0.7***	0	3.7	5.5	5.1	0.7	0.1	7.9	9.9	8.9	2.4	0.3
	1200	3.4	*14.9*	*2*	0	0	*5.7*	*18.5*	***2.3***	0.1	0	7.1	11.4	2.8	0.5	0
T4	600	12.6	***19***	2.7	0.1	0	16	13.2	2.4	0.1	0	18.8	13.4	3.3	0.3	0
	1200	8	5.4	0.2	0	0	***10.7***	5.7	0.2	0	0	8.6	4.8	0.4	0	0
T5	600	17.1	5.6	0.8	0	0	17.2	4.3	0.7	0	0	17.8	4.4	0.9	0	0
	1200	5.3	4	0.1	0	0	5.1	4.2	0.1	0	0	4.2	3.6	0.1	0	0
T6	600	10.4	5.1	1.2	0	0	11.2	4.2	1.1	0	0	11.7	4.1	1.3	0.1	0
	1200	3.3	4.9	0.1	0	0	3.4	5.2	0.1	0	0	2.9	4.2	0.2	0	0

Table 15: TITE-comb-BOIN12 Assignments 2

	Agent W_1 (mg)	A7 Agent W_2 (kBq/kg) 50	75	100	125	150	A8 50	75	100	125	150
T1	600	*1.9*	*4.9*	*8.8*	*0.4*	*0*	*1.8*	*4.8*	*8.8*	*0.3*	*0*
	1200	*2.6*	*16.3*	***2.8***	*0*	*0*	*3*	*16.1*	***2.8***	*0*	*0*
T2	600	*16.4*	*14.4*	***2.8***	*0.1*	0	*16.5*	*14.5*	***2.9***	*0.1*	0
	1200	3.5	4.5	0.1	0	0	3.5	4.5	0.1	0	0
T3	600	*3.1*	*6.5*	*8.1*	*0.2*	0	*2.9*	*6.3*	*8.1*	*0.1*	0
	1200	*3.4*	*15.4*	***1.3***	0	0	*3.8*	*15.4*	***1.4***	0	0
T4	600	*16.9*	***12.8***	1.5	0	0	*16.7*	***12.7***	1.5	0	0
	1200	*6.1*	5.2	0.1	0	0	*6.7*	5.2	0.1	0	0
T5	600	***17.9***	4	0.6	0	0	***17.9***	4	0.6	0	0
	1200	3.7	4.1	0	0	0	3.8	4.1	0	0	0
T6	600	11.2	4.4	1	0	0	11.2	4.4	1	0	0
	1200	2.8	5.2	0.1	0	0	2.8	5.2	0.1	0	0

Table 16: TITE-comb-BOIN12 Assignments 3

	Agent W_1 (mg)	A1 Agent W_2 (kBq/kg) 50	75	100	125	150	A2 50	75	100	125	150	A3 50	75	100	125	150
T1	600	*4.5*	*6.5*	*8*	*7.3*	*2.2*	*6.4*	*7.1*	*8.7*	*5.6*	*2.5*	5.6	5.9	6.7	*6.8*	*4.4*
	1200	*4.9*	*10.8*	*7.6*	*4.8*	***0.5***	*7.3*	*8.9*	***6.8***	*3.5*	***0.8***	5.9	7.2	*7.4*	***6.2***	***2.1***
T2	600	*13.6*	*14.5*	*9.4*	***4***	0.2	*15.9*	*13.4*	*9.1*	***2.8***	0.2	14.4	12.4	9.2	***3.9***	0.8
	1200	6.1	5.7	2	0.3	0	6.4	5.4	1.8	0.3	0	6.8	5.3	2.9	0.7	0.1
T3	600	*6.2*	*8.7*	*9*	***5.6***	0.8	*8.6*	*8.8*	*9.3*	*4.2*	0.8	7.8	8.1	8.3	*6.2*	2
	1200	*6.6*	*12.4*	*6.6*	1.4	0.1	*8.9*	*10*	***5.7***	1.3	0.1	8	8.9	***6.8***	2.9	0.5
T4	600	*14.7*	***16***	5.7	0.6	0	*17*	*13.8*	5.1	0.4	0	16.8	13.6	5.7	0.9	0.1
	1200	10.2	6.9	0.8	0	0	***11.2***	6	0.7	0	0	11.1	6.1	1.3	0.1	0
T5	600	***17.3***	4.9	0.9	0	0	***17.1***	4.7	0.9	0	0	16.5	4.7	1.3	0.1	0
	1200	5.2	4.3	0.1	0	0	4.7	4	0.1	0	0	5.6	3.7	0.2	0	0
T6	600	11.3	4.2	1.1	0.1	0	11.4	4	1.2	0.1	0	10.8	4.1	1.5	0.2	0
	1200	3.2	5.2	0.2	0	0	3.2	4.6	0.1	0	0	3.6	3.9	0.3	0	0

Table 17: TITE-comb-BOIN12 Assignments 1 (Alternative BOIN Utility)

	Agent W_1 (mg)	A4 Agent W_2 (kBq/kg) 50	75	100	125	150	A5 50	75	100	125	150	A6 50	75	100	125	150
T1	600	4.1	*6.3*	*8.9*	*8.5*	***4.5***	5	5.4	5.7	4.3	1.9	7.8	7.4	7.2	5.8	3.4
	1200	4.2	*7.2*	*7.4*	*5.9*	***1.4***	*6.6*	*10*	*10.2*	*7*	***1.4***	7.4	7.1	5.8	3.8	***2.3***
T2	600	11.1	*14.6*	*11.5*	**4.7**	0.6	14.6	12.7	8.6	3.2	0.5	15.7	13	8.5	3.5	0.8
	1200	5.6	5.3	2.5	0.5	0	7.2	6.1	2.9	0.7	0.1	6.6	5.1	2.4	0.8	0.2
T3	600	5.9	*8.9*	*10.9*	***7.4***	1.6	7.1	7.6	6.9	3.6	0.9	10	9.6	8.5	5.1	1.6
	1200	5.9	*9*	*6.6*	2.5	0.3	*8.7*	*12.5*	***8.6***	2.6	0.2	8.9	8.2	5.2	2.1	0.6
T4	600	13.7	***17.3***	6.5	0.8	0.1	15.7	13.2	5.1	0.7	0.1	18.1	13.6	5.3	0.9	0.1
	1200	9.3	6.4	1.1	0.1	0	***12.2***	7	1.2	0.1	0	10.5	5.7	1.1	0.2	0
T5	600	15.9	5.5	1.2	0.1	0	16.5	4.9	1.1	0.1	0	16.8	4.7	1.3	0.1	0
	1200	5.5	3.9	0.2	0	0	5.8	4	0.2	0	0	5	3.5	0.2	0	0
T6	600	10.3	4.8	1.6	0.2	0	11.1	4.1	1.3	0.1	0	11.2	4	1.5	0.2	0
	1200	3.4	4.2	0.2	0	0	3.7	4.4	0.3	0	0	3.4	3.6	0.3	0	0

Table 18: TITE-comb-BOIN12 Assignments 2 (Alternative BOIN Utility)

	Agent W_1 (mg)	A7 Agent W_2 (kBq/kg) 50	75	100	125	150	A8 50	75	100	125	150
T1	600	*4.9*	*6.9*	*10.1*	*5.9*	*2.1*	*5*	*7.1*	*10.5*	*4.7*	*1.6*
	1200	*5.2*	*9.2*	***8.5***	*4*	*0.8*	*6.3*	*9.4*	***9***	*3.3*	*0.8*
T2	600	*13.3*	*14.3*	***10.9***	*3.2*	0.3	*13.3*	*14.3*	***11***	*2.6*	0.2
	1200	6	5.4	2.1	0.3	0	6.4	5.4	2.2	0.4	0
T3	600	*6.5*	*9*	*11.2*	*4.7*	0.8	*6.4*	*9*	*11.3*	*3.6*	0.6
	1200	*6.7*	*10.4*	***6.8***	1.4	0.1	*7.9*	*10.4*	***6.8***	1.3	0.2
T4	600	*14.9*	***15.8***	6.2	0.4	0	*14.6*	***15.3***	5.9	0.4	0
	1200	*10.3*	6.4	0.7	0.1	0	*11.4*	6.3	0.7	0.1	0
T5	600	***17.1***	4.9	1	0.1	0	***16.9***	4.9	1	0.1	0
	1200	5.1	4.1	0.1	0	0	5.3	4.1	0.1	0	0
T6	600	11.3	4.3	1.3	0.1	0	11.2	4.3	1.3	0.1	0
	1200	3.3	4.6	0.2	0	0	3.5	4.6	0.2	0	0

Table 19: TITE-comb-BOIN12 Assignments 3 (Alternative BOIN Utility)

8 All Recommendations

Acceptable dose combinations are highlighted in *italics*, good dose combinations are underlined and correct dose combinations are highlighted in **boldface**.

As well as percentage recommendations for each of the dose combinations, we also include the percentage of recommendations of recommending no dose combination, given in the column labelled 'N'. The top cell indicates the percentage of simulations recommending the highest dose is too safe, and the bottom cell indicates the percentage of simulations recommending no admissible dose, or stopping for safety.

	Agent W_1 (mg)	A1						A2 Agent W_2 (KBq/kg)						A3					
		50	75	100	125	150	N	50	75	100	125	150	N	50	75	100	125	150	N
T1	600	*0*	*0*	*0*	*0*	*0*	11.5	*0*	*0*	*0*	*0*	*0*	11.5	0	0	0	*0*	*0*	10.5
	1200	*1.8*	*9.9*	*26.6*	*38.8*	***11.4***	0	*1.8*	*9.6*	***26.6***	*38.7*	*11.7*	0.1	0.4	7.4	*26.3*	*38.1*	**10.1**	7.2
T2	600	*11.4*	*15.5*	20.4	***14.1***	2.9	0	*17.4*	*17.1*	***22***	*13.8*	3.6	0	0.2	0.5	3.4	***3.5***	0.7	0
	1200	16.3	1.8	0.2	0	0	17.3	19.2	2.1	0.1	0	0	4.7	2.9	1.2	0.1	0	0	87.4
T3	600	*0*	*0*	0	***0.1***	0	0.3	*0*	*0*	***0***	*0*	0	0.4	0	0	0	***0***	0	0.5
	1200	*7*	*42.7*	44.4	4.7	0.1	0.7	*6.8*	*43.1*	*44.2*	4.9	0.1	0.5	1.1	26	*37.4*	3.9	0	31.1
T4	600	*7.8*	***10.1***	2.3	0	0	0	***10.2***	*11.2*	2.6	0.1	0	0	0	0.2	0.3	0.1	0	0
	1200	67.4	4.3	0.1	0	0	8	*68*	3.8	0	0	0	4.1	6.4	0.7	0.1	0	0	92.2
T5	600	***32.3***	2.1	0.3	0	0	0	***46.7***	1.5	0.2	0	0	0	1.2	0.1	0	0	0	0
	1200	26	0.8	0	0	0	38.6	28	0.9	0	0	0	22.6	9.8	1.2	0	0	0	***87.7***
T6	600	21.4	2	0.1	0.1	0	0	24.6	1.8	0.3	0	0	0	0.5	0.4	0.1	0	0	0
	1200	27.6	3.9	0.1	0	0	***44.8***	23.1	3.6	0.1	0	0	***46.4***	7	3.3	0	0	0	***88.7***

Table 20: Joint TITE-BLRM Recommendations 1

	Agent W_1 (mg)	A4						A5 Agent W_2 (KBq/kg)						A6					
		50	75	100	125	150	N	50	75	100	125	150	N	50	75	100	125	150	N
T1	600	0	*0*	*0*	*0*	*0*	11	0	0	0	0	0	10.6	0	0	0	0	0	8.4
	1200	0.5	*9.9*	*25.1*	*40.9*	***10.7***	1.9	*1.2*	*9.5*	*25.1*	*41.1*	***11.8***	0.7	0.2	1.4	5.2	7.1	***3***	74.7
T2	600	0.4	*8.8*	*12.3*	***7.8***	1.7	0	1.8	8.3	12	10.5	3.3	0	0.8	1.4	1.4	1.3	0.7	0
	1200	9.9	1.7	0.2	0	0	57.1	12.5	1.7	0.1	0	0	***49.8***	3.7	0.7	0	0	0	***89.9***
T3	600	0	*0*	*0*	***0***	0	0.5	0	0	0	0.1	0	0.2	0	0	0	0	0	0.3
	1200	1.9	*42.8*	*44.2*	4.2	0.1	6.3	*4.9*	*41.6*	***46.8***	4.2	0.1	2.1	0.8	7.2	11	1.6	0	***79.2***
T4	600	0.4	***3.4***	1.1	0	0	0	0.9	5.2	2.3	0.3	0	0	0.4	0.8	0.2	0	0	0
	1200	18.4	2.3	0.1	0	0	74.3	***53.5***	3.7	0.1	0	0	34	6.4	1.3	0	0	0	***90.9***
T5	600	2	1.4	0.2	0.1	0	0	10.9	1.9	0.1	0	0	0	5.3	0.2	0	0	0	0
	1200	15	0.9	0	0	0	***80.4***	26.3	1	0	0	0	***59.8***	12.2	0.8	0	0	0	***81.5***
T6	600	1.7	1.8	0.2	0	0.1	0	4.4	1.3	0.5	0	0	0	1.8	0.1	0	0.1	0	0
	1200	11.8	3.5	0.1	0	0	***80.8***	19.6	3.7	0	0	0	***70.5***	5.9	2.5	0	0	0	***89.6***

Table 21: Joint TITE-BLRM Recommendations 2

	Agent W_1 (mg)	A7 Agent W_2 (kBq/kg)						A8					
		50	75	100	125	150	N	50	75	100	125	150	N
T1	600	*0*	*0*	*0*	*0*	*0*	11.3	*0*	*0*	*0*	*0*	*0*	11.2
	1200	*1.8*	*9.9*	***25.9***	*39.7*	*11.4*	0	*1.8*	*9.8*	***25.8***	*40.6*	*10.8*	0
T2	600	*11.2*	*16*	***19.6***	*13.5*	3	0	*12.2*	*18.2*	***21.5***	*15.3*	3.4	0
	1200	15.8	2	0.1	0	0	18.7	18.7	2.1	0.1	0	0	8.5
T3	600	*0*	*0*	*0*	*0*	0	0.4	*0*	*0*	*0*	*0*	0	0.5
	1200	*7*	*43.5*	***43.7***	4.5	0.1	0.8	*7.2*	*43.9*	***43.7***	4.1	0.1	0.5
T4	600	*7.5*	***10.8***	2.8	0.1	0	0	*7.7*	***11.2***	2.4	0	0	0
	1200	*65.2*	4.3	0	0	0	9.3	*66.8*	4.3	0	0	0	7.6
T5	600	***28.5***	1.6	0	0	0	0	***35.1***	2.3	0.1	0	0	0
	1200	22.8	0.6	0	0	0	46.5	27.9	0.8	0	0	0	33.9
T6	600	20.2	2	0.1	0	0.1	0	24.6	2.7	0.3	0.1	0	0
	1200	29	4.1	0.1	0	0	***44.4***	30.3	4.4	0.1	0	0	***37.5***

Table 22: Joint-TITE-BLRM Recommendations 3

	Agent W_1 (mg)	A1						A2 Agent W_2 (KBq/kg)						A3					
		50	75	100	125	150	N	50	75	100	125	150	N	50	75	100	125	150	N
T1	600	*0*	*0*	*0.5*	*3.8*	*20.5*	48.1	*0*	*0*	*0.5*	*3.2*	*18.3*	50.5	0	0	0	*3.4*	*19.3*	45.7
	1200	*0*	*1.7*	*5.9*	*16.6*	***2.7***	0.2	*0.4*	*2.8*	***6.2***	*14.5*	*3.3*	0.3	0.2	3.4	*6.8*	*13.5*	**2.4**	5.3
T2	600	*0.2*	*8.3*	33.5	***36.3***	8.3	0	*0.3*	*8.8*	***32.5***	*36.2*	8.7	0	0	0.3	11.3	***18.2***	4.7	0.1
	1200	2.8	2.1	0.4	0	0	8.1	3.8	1.3	0.3	0	0	8.1	0.9	0.9	0.5	0.1	0	62.9
T3	600	*0*	*0.5*	4.8	***30.8***	6.6	5.1	*0*	*0.5*	***5.7***	*29.2*	7.4	5.3	0	0	2.7	***23.2***	5.6	5.2
	1200	*0.5*	*14.1*	30	6.8	0	0.8	*0.8*	*13.9*	*29.4*	7.1	0.1	0.6	0.6	10	*24.1*	6.9	0	21.5
T4	600	*0.9*	***42.3***	13.1	1.1	0.1	0	***0.9***	*38.2*	13.8	0.7	0.1	0	0	2.3	8.4	0.4	0.2	0.1
	1200	24.8	4.6	0	0	0	13.1	*29.7*	4.7	0	0	0	11.9	5.2	3.3	0	0	0	80
T5	600	***15.3***	6.2	1	0	0	0	***30.9***	7.4	1	0.1	0	0	0	0.5	0.2	0	0	0
	1200	7.6	0	0	0	0	69.8	8.6	0	0	0	0	52	0.9	0.1	0	0	0	***98.3***
T6	600	10.4	9.3	1.2	0	0	0	23.2	11.8	1.4	0.1	0	0	0	0.5	0	0	0	0
	1200	8.8	1	0	0	0	***69.2***	9.6	1.5	0	0	0	***52.4***	0.7	0.4	0	0	0	***98.4***

Table 23: Joint TITE-POCRM Recommendations 1

	Agent W_1 (mg)	A4						A5 Agent W_2 (KBq/kg)						A6					
		50	75	100	125	150	N	50	75	100	125	150	N	50	75	100	125	150	N
T1	600	0	*0*	*0.6*	*4.2*	*26.4*	47.3	0	0	0.2	0.5	4.5	51.4	0	0	0	0.7	2.3	34.4
	1200	0.1	*1*	*5.5*	*11.6*	***2.7***	0.6	*0.4*	*4.5*	*11.4*	*21.2*	***4.4***	1.5	0.1	0.8	1.4	1.5	***0.6***	58.1
T2	600	0	*7.9*	*33.1*	***34.3***	7.4	0	0.1	1	12	20.7	6.4	0.1	0	0.1	2.9	4.6	2.5	0.1
	1200	1.1	1.8	0.8	0	0	13.6	4.2	1.2	0.5	0	0	***53.7***	0.6	0.1	0.1	0	0	***89***
T3	600	0	*0.4*	*7.1*	***36.6***	7.5	5.4	0	0.1	1.5	10.4	2.6	5.5	0	0	0.6	4.7	2.3	4.3
	1200	0	*7.9*	*25.7*	7.7	0.1	1.6	*1.4*	*30.6*	***37***	6	0.1	4.8	0.3	2.8	4.5	2.4	0	***78.2***
T4	600	0	***29.8***	12.8	0.7	0.3	0	0	4.7	6.1	1.1	0	0	0	1.1	1.9	0	0	0
	1200	8.8	3.7	0	0	0	43.9	***17***	1.7	0	0	0	69.4	1.9	0.8	0	0	0	***94.3***
T5	600	0.2	6.8	0.3	0	0	0	1.6	2.2	0.3	0.1	0	0	1.1	2	0.9	0	0	0
	1200	4.5	0.1	0	0	0	***88.1***	6.2	0.1	0	0	0	***89.5***	2.6	0.2	0	0	0	***93.2***
T6	600	0.4	11.4	0.8	0	0	0	0.3	2	0.6	0.2	0.1	0	0.2	1.3	0.2	0.2	0	0
	1200	4.2	1.7	0	0	0	***81.5***	5.3	0.9	0	0	0	***90.6***	1.5	1.1	0	0	0	***95.5***

Table 24: Joint TITE-POCRM Recommendations 2

	Agent W_1 (mg)	A7						A8					
		Agent W_2 (kBq/kg)											
		50	75	100	125	150	N	50	75	100	125	150	N
T1	600	*0*	*0*	*0.8*	*2.7*	*16.7*	50.1	*0*	*0*	*0.7*	*1.3*	*8.8*	52.6
	1200	*0.2*	*2.4*	***8.1***	*15.2*	*3.5*	0.3	*0.6*	*2.8*	***9.9***	*19.2*	*3.8*	0.3
T2	600	*0.3*	*7.1*	***34.8***	*35.7*	8.7	0.1	*0.1*	*8.2*	***32.7***	*37.2*	7.4	0.1
	1200	2.7	2	0.8	0	0	7.8	3.4	2.1	0.5	0	0	8.3
T3	600	*0*	*0.3*	*6.9*	*27.1*	7.1	5.6	*0*	*0.3*	*5.9*	*20.5*	3.4	5.5
	1200	*0.6*	*12.5*	***32.1***	7.1	0.1	0.6	*0.9*	*17.9*	***38***	6.5	0.2	0.9
T4	600	*0.8*	***43.4***	11.9	1.1	0	0	*0.8*	***38.6***	12.5	1	0	0
	1200	*25.5*	4	0	0	0	13.3	*29.8*	4.1	0	0	0	13.2
T5	600	***14.1***	6.6	0.5	0	0	0	***20.1***	7	0.8	0	0	0
	1200	8.1	0	0	0	0	70.7	9.3	0	0	0	0	62.8
T6	600	13.4	10.9	1.2	0	0	0	11.4	8.5	1.2	0.1	0	0
	1200	9.3	1.4	0	0	0	***63.8***	6.9	0.7	0	0	0	***71.1***

Table 25: Joint-TITE-POCRM Recommendations 3

	Agent W_1 (mg)	A1						A2						A3					
		Agent W_2 (KBq/kg)																	
		50	75	100	125	150	N	50	75	100	125	150	N	50	75	100	125	150	N
T1	600	*4.5*	*11.6*	*14.5*	*1*	*0*	0.1	*6.4*	*12.3*	*20.4*	*1*	*0*	0	3.9	7.7	15.6	*7.6*	*0.7*	0.2
	1200	*7*	*55.3*	*6*	*0*	***0***	0	*8.3*	*45.1*	***6.5***	*0*	*0*	0	9.5	29.6	*20.2*	*2.5*	**0**	2.5
T2	600	*45.1*	*43.5*	5.8	***0.5***	0	0	*55.8*	*34.5*	***5.5***	*0.4*	0	0	23.8	20.2	22.7	***5.7***	0.3	0
	1200	1.7	0.4	0	0	0	3	1.1	0.4	0	0	0	2.3	2.9	1.1	0.7	0	0	22.6
T3	600	*7.1*	*16*	13.9	***0.5***	0	0	*10.5*	*16.3*	***19.3***	*0.4*	0	0	5.6	10.4	17.4	***7.3***	0	0
	1200	*9.1*	*51.5*	1.8	0.1	0	0	*10.4*	*41.2*	*1.9*	0	0	0	11.9	34	*7.5*	0.2	0	5.7
T4	600	*46.5*	***35.8***	1.1	0.1	0	0	***55.4***	*28.9*	1	0	0	0	25.4	20.8	4.6	0.6	0	0
	1200	10.5	2.5	0	0	0	3.5	*10.3*	1.8	0	0	0	2.6	14.9	4	0.5	0	0	29.2
T5	600	***44.9***	1.5	0	0	0	0	***45.9***	1	0	0	0	0	36.8	1.4	0	0	0	0
	1200	3.2	0.5	0	0	0	49.9	2.6	0.5	0	0	0	50	3.2	0.8	0	0	0	***57.8***
T6	600	13.6	3.3	0.1	0	0	0	14.1	3	0	0	0	0	10.2	1.7	0.1	0	0	0
	1200	1.7	4.2	0	0	0	***77.1***	1.6	3.3	0	0	0	***78***	1.8	2.7	0	0	0	***83.5***

Table 26: TITE-comb-BOIN12 Recommendations 1

	Agent W_1 (mg)	A4 50	75	100	125	150	N	A5 50	75	100	125	150	N	A6 50	75	100	125	150	N
		Agent W_2 (KBq/kg)																	
T1	600	0.3	*17*	*25.7*	*2.8*	*0.1*	0	2.6	6.6	7.3	1.6	0.3	0.1	12.4	13.9	15.2	7.7	2.2	1.8
	1200	3.5	*38*	*11.8*	*0.2*	***0***	0.6	*12.1*	*54.9*	*13.2*	*1.1*	***0***	0.2	13.1	18.3	10.8	1.5	***0.2***	2.9
T2	600	12.7	*58.4*	*19.4*	***1.2***	0	0	29.7	33.2	16.7	2.6	0.2	0	36.3	25.2	15	2.6	0.3	0
	1200	1.8	0.2	0	0	0	6.3	2.9	0.8	0.3	0.1	0	***13.5***	2.8	0.5	0	0.1	0	***17.2***
T3	600	0.8	*22.6*	*26*	***2***	0	0	4.4	8.7	8.3	1.4	0	0	15.2	18.1	17.2	4.4	0.4	0
	1200	3.7	*40.4*	*3.4*	0.1	0	1	*16.1*	*56*	***4.6***	0.3	0	0.2	15.2	18.6	3.9	1.1	0	***5.9***
T4	600	15.6	***54.7***	2.8	0.1	0	0	29.7	26.3	2.3	0.4	0	0	37.6	24.3	2.8	0.2	0	0
	1200	10.4	2.2	0.1	0	0	14.1	***24.6***	3.2	0.1	0	0	13.4	11.8	2.3	0.1	0	0	***20.9***
T5	600	34.2	2.7	0.1	0	0	0	36.7	1.3	0	0	0	0	39.8	2.1	0	0	0	0
	1200	4.1	0.7	0	0	0	***58.2***	5.5	0.6	0	0	0	***55.9***	2.8	0.3	0	0	0	***55***
T6	600	9.2	3.8	0	0	0	0	12.1	2.1	0	0	0	0	13.2	2.3	0	0.1	0	0
	1200	2.2	2.7	0	0	0	***82.1***	2.4	3.2	0	0	0	***80.2***	0.8	1.6	0	0	0	***82***

Table 27: TITE-comb-BOIN12 Recommendations 2

	Agent W_1 (mg)	A7 50	75	100	125	150	N	A8 50	75	100	125	150	N
		Agent W_2 (kBq/kg)											
T1	600	*4.3*	*12.7*	*23.6*	*0.8*	*0*	0	*3.8*	*12.2*	*23.5*	*0.5*	*0*	0
	1200	*6.9*	*44.1*	**7.6**	*0*	*0*	0	*8.9*	*43.2*	**7.9**	*0*	*0*	0
T2	600	*44.3*	*43*	***7.4***	*0.3*	0	0	*44.5*	*43.1*	**7.7**	*0.2*	0	0
	1200	1.8	0.3	0	0	0	2.9	1.4	0.3	0	0	0	2.8
T3	600	*7.3*	*17.5*	*21.5*	*0.5*	0	0	*6.6*	*16.7*	*21.7*	*0.2*	0	0
	1200	*9.1*	*41.7*	***2.4***	0	0	0	*11*	*41.4*	***2.4***	0	0	0
T4	600	*47.1*	**35.5**	1.1	0	0	0	*45.2*	**35.5**	1.1	0	0	0
	1200	*10.8*	2.1	0	0	0	3.4	*12.9*	2.1	0	0	0	3.2
T5	600	***44.3***	1.6	0	0	0	0	***44.2***	1.7	0	0	0	0
	1200	3.3	0.5	0	0	0	50.3	3.4	0.5	0	0	0	50.2
T6	600	13.5	3.1	0.1	0	0	0	13.2	3.1	0.1	0	0	0
	1200	1.9	3.4	0	0	0	***78***	1.8	3.4	0	0	0	***78.4***

Table 28: TITE-comb-BOIN12 Recommendations 3

	Agent W_1 (mg)	A1						A2						A3					
		Agent W_2 (kBq/kg)																	
		50	75	100	125	150	N	50	75	100	125	150	N	50	75	100	125	150	N
T1	600	*13.9*	*10*	*12.4*	*11.5*	*3.7*	2.4	*15*	*10.4*	*11.8*	*7.8*	*3.6*	3.1	14.1	7.2	8.4	*9.1*	*7.2*	10
	1200	*12.1*	*12.8*	*13.1*	*7.3*	***0.3***	0.5	*13.6*	*13.1*	***14.7***	*5.5*	***1.3***	0.1	10.6	9.5	*11.3*	***7.6***	***4.4***	0.6
T2	600	*37.8*	*26.3*	*17.5*	***6.5***	0.5	0	*36.4*	*28.2*	*18.6*	***5.1***	0.3	0	22.4	18.1	18.7	***8.7***	0.9	0
	1200	4.1	2.3	1.2	0	0	3.8	3.7	3	1.2	0	0	3.5	3.5	3.3	1.4	0.5	0	22.5
T3	600	*20.5*	*13*	*11*	***9.2***	0.8	0	*21.2*	*13.1*	*13.8*	*7.2*	0.8	0.1	18.3	9.9	12.9	*11.9*	1	0.7
	1200	*18.6*	*14.9*	*8.8*	2.3	0.1	0.8	*16.6*	*16.4*	***8.1***	1.9	0.2	0.6	14.6	14.7	***9.1***	3.7	0.7	2.5
T4	600	*39.4*	***28.5***	5.1	0.2	0	0	*42.9*	*25*	4.6	0.1	0	0	21.1	15.7	7	0.6	0	0
	1200	16.7	4.3	0.7	0	0	5.1	***17.7***	4.3	0.5	0.1	0	4.8	13.2	5.7	1.1	0.1	0	***35.5***
T5	600	***39.5***	1.1	0.4	0	0	0	***39.6***	1.3	0.5	0	0	0	30.2	0.4	0.6	0	0	0
	1200	2.9	0.8	0.2	0	0	55.1	2.6	0.7	0.1	0	0	55.2	3.3	0.5	0.1	0	0	***64.9***
T6	600	13.3	1.9	0.2	0	0	0	13.5	1.6	0.1	0	0	0	9	0.9	0.4	0	0	0
	1200	1	2.9	0.2	0	0	***80.5***	1	1.7	0	0	0	***82.1***	1.2	0.5	0.2	0	0	***87.8***

Table 29: TITE-comb-BOIN12 Recommendations 1 (Alternative BOIN Utility)

	Agent W_1 (mg)	A4						A5						A6					
		Agent W_2 (kBq/kg)																	
		50	75	100	125	150	N	50	75	100	125	150	N	50	75	100	125	150	N
T1	600	14.1	*7.4*	*9.1*	*10.2*	***6.5***	5.6	14.7	9.9	9.9	10	4	6.4	12.7	8	9.5	8.9	6.8	9.9
	1200	12.4	*12.4*	*12.2*	*7.3*	***2.3***	0.5	*10.8*	*8.2*	*13.8*	*10.6*	***1.3***	0.4	12.2	9.8	11.9	5.6	***3.5***	1.2
T2	600	29	*27.8*	*21.7*	**6.3**	1.2	0	31	20.7	21.8	7.3	1	0	28.5	18.5	18.2	8.7	1.4	0
	1200	3.6	2.7	1	0.1	0	6.6	3.8	1.3	1	0.4	0.1	11.6	2.9	3.2	2.3	0.6	0.2	***15.5***
T3	600	23.9	*10.6*	*11.4*	***10.3***	0.6	0.4	22.2	14.5	14.7	8.2	1.4	0	16.2	12.3	10.9	11.1	1.5	0.5
	1200	17.8	*13.1*	*7.4*	2.7	0.2	1.6	*14.9*	*9.6*	***9.6***	3.2	0.1	1.6	15.2	16.6	8.8	3.6	0.9	***2.4***
T4	600	24.9	***35.7***	4.6	0.4	0	0	31.9	23.9	7	1	0	0	31.5	17.8	6.4	1	0	0
	1200	14.3	4.3	0.7	0.1	0	15	***16.9***	3.7	1	0.3	0	14.3	12.5	5.2	2	0.1	0	***23.5***
T5	600	28.6	2	0.3	0	0	0	32.5	1.5	0.6	0	0	0	33.5	1	0.7	0	0	0
	1200	3.2	0.7	0	0	0	***65.2***	4.5	0.6	0.1	0	0	***60.2***	2.8	0.5	0.2	0	0	***61.3***
T6	600	8.5	2.2	0.1	0.1	0	0	11.8	1.6	0.2	0.1	0	0	11	1.1	0.4	0	0	0
	1200	1.7	1	0	0	0	***86.4***	2.4	1.1	0.1	0	0	***82.7***	1.2	1.1	0.2	0	0	***85***

Table 30: TITE-comb-BOIN12 Recommendations 2 (Alternative BOIN Utility)

		A7						A8					
	Agent W_1 (mg)	Agent W_2 (kBq/kg)											
		50	75	100	125	150	N	50	75	100	125	150	N
T1	600	*12.8*	*9.5*	*13.6*	*11.4*	*4.2*	3	*15.5*	*10.4*	*11.4*	*9.4*	*3.7*	2.9
	1200	*12.1*	*12*	***14.4***	*5.5*	*1.2*	0.3	*14.1*	*12.9*	***13.3***	*4.6*	*1.3*	0.5
T2	600	*36.3*	*26.9*	***18.1***	*5.1*	0.3	0	*36.1*	*26*	***18.9***	*6.1*	0.9	0
	1200	4.4	3.1	1.7	0	0	4.1	4	2.9	1	0	0	4.1
T3	600	*20.5*	*12.9*	*12.7*	*7.7*	0.9	0.1	*21.3*	*13.4*	*13.1*	*7*	1.2	0
	1200	*17.7*	*14.5*	***9.5***	2.2	0.2	1.1	*17*	*13.6*	***9.6***	2.2	0.3	1.3
T4	600	*39*	***28.8***	4.8	0.2	0	0	*38.9*	***27.4***	4.7	0.3	0	0
	1200	*17.5*	3.7	0.9	0	0	5.1	*18.1*	4.3	0.8	0.1	0	5.4
T5	600	***38.9***	1.1	0.5	0	0	0	***37.8***	1.5	0.6	0	0	0
	1200	2.4	0.8	0.2	0	0	56.1	2.8	0.8	0.2	0	0	56.3
T6	600	12.6	1.9	0.5	0	0	0	12.7	1.7	0.6	0	0	0
	1200	0.9	2	0.2	0	0	***81.9***	1.3	2.1	0.2	0	0	***81.4***

Table 31: TITE-comb-BOIN12 Recommendations 3 (Alternative BOIN Utility)

References

[1] Helen Barnett, Oliver Boix, Dimitris Kontos, and Thomas Jaki. Joint tite-crm: A design for dose finding studies for therapies with late-onset safety and activity outcomes. *Statistics in Biopharmaceutical Research*, 0(0):1–12, 2024. 3, 6

[2] Ruitao Lin and Guosheng Yin. Bayesian optimal interval design for dose finding in drug-combination trials. *Statistical methods in medical research*, 26(5):2155–2167, 2017. 1

[3] Mengyi Lu, Jingyi Zhang, Ying Yuan, and Ruitao Lin. Comb-boin12: A utility-based bayesian optimal interval design for dose optimization in cancer drug-combination trials. *Statistics in Biopharmaceutical Research*, 17(2):266–276, 2025. 1

[4] Pavel Mozgunov, Rochelle Knight, Helen Barnett, and Thomas Jaki. Using an interaction parameter in model-based phase I trials for combination treatments? A simulation study. *International Journal of Environmental Research and Public Health*, 18(1):1–19, 2021. 3

[5] Pavel Mozgunov, Xavier Paoletti, and Thomas Jaki. A benchmark for dose-finding studies with unknown ordering. *Biostatistics*, 23(3):721–737, 01 2021. 13

[6] Nolan A Wages, Mark R Conaway, and John O'Quigley. Continual reassessment method for partial ordering. *Biometrics*, 67(4):1555–1563, 2011. 3

[7] Nolan A. Wages and Nikole Varhegyi. pocrm: An r-package for phase i trials of combinations of agents. *Computer Methods and Programs in Biomedicine*, 112(1):211–218, 2013. 4

[8] Yanhong Zhou, Ruitao Lin, J. Jack Lee, Daniel Li, Li Wang, Ruobing Li, and Ying Yuan. Tite-boin12: A bayesian phase i/ii trial design to find the optimal biological dose with late-onset toxicity and efficacy. *Statistics in Medicine*, 41(11):1918–1931, 2022. 1, 7
